# *yippee like 3 (ypel3)* is a novel gene required for myelinating and perineurial glia development

**DOI:** 10.1371/journal.pgen.1008841

**Published:** 2020-06-16

**Authors:** Bernardo Blanco-Sánchez, Aurélie Clément, Sara J. Stednitz, Jennifer Kyle, Judy L. Peirce, Marcie McFadden, Jeremy Wegner, Jennifer B. Phillips, Ellen Macnamara, Yan Huang, David R. Adams, Camilo Toro, William A. Gahl, May Christine V. Malicdan, Cynthia J. Tifft, Erika M. Zink, Kent J. Bloodsworth, Kelly G. Stratton, Undiagnosed Diseases Network, David M. Koeller, Thomas O. Metz, Philip Washbourne, Monte Westerfield

**Affiliations:** 1 Institute of Neuroscience, University of Oregon, Eugene, Oregon, United States of America; 2 Pacific Northwest National Laboratory, Richland, Washington, United States of America; 3 National Institutes of Health Undiagnosed Diseases Program, Common Fund, Office of the Director, National Institutes of Health, Bethesda, Maryland, United States of America; 4 Office of the Clinical Director, National Human Genome Research Institute, National Institutes of Health, Bethesda, Maryland, United States of America; 5 Section of Human Biochemical Genetics, Medical Genetics Branch, National Human Genome Research Institute, National Institutes of Health, Bethesda, Maryland, United States of America; 6 Molecular and Medical Genetics, School of Medicine, Oregon Health and Science University, Portland, Oregon, United States of America; Fred Hutchinson Cancer Research Center, UNITED STATES

## Abstract

Hypomyelination, a neurological condition characterized by decreased production of myelin sheets by glial cells, often has no known etiology. Elucidating the genetic causes of hypomyelination provides a better understanding of myelination, as well as means to diagnose, council, and treat patients. Here, we present evidence that *YIPPEE LIKE 3 (YPEL3)*, a gene whose developmental role was previously unknown, is required for central and peripheral glial cell development. We identified a child with a constellation of clinical features including cerebral hypomyelination, abnormal peripheral nerve conduction, hypotonia, areflexia, and hypertrophic peripheral nerves. Exome and genome sequencing revealed *a de novo* mutation that creates a frameshift in the open reading frame of *YPEL3*, leading to an early stop codon. We used zebrafish as a model system to validate that *YPEL3* mutations are causative of neuropathy. We found that *ypel3* is expressed in the zebrafish central and peripheral nervous system. Using CRISPR/Cas9 technology, we created zebrafish mutants carrying a genomic lesion similar to that of the patient. Our analysis revealed that Ypel3 is required for development of oligodendrocyte precursor cells, timely exit of the perineurial glial precursors from the central nervous system (CNS), formation of the perineurium, and Schwann cell maturation. Consistent with these observations, zebrafish *ypel3* mutants have metabolomic signatures characteristic of oligodendrocyte and Schwann cell differentiation defects, show decreased levels of Myelin basic protein in the central and peripheral nervous system, and develop defasciculated peripheral nerves. Locomotion defects were observed in adult zebrafish *ypel3* mutants. These studies demonstrate that Ypel3 is a novel gene required for perineurial cell development and glial myelination.

## Author summary

The study of congenital rare diseases allows the identification of new gene variants involved in development. Genetic and sequence analysis of a patient with a novel complex phenotype including hypomyelination, enlarged peripheral nerves, and motor defects implicated a *de novo* mutation in the *YPEL3* gene as potentially causative. *YPEL3* had not previously been associated with disease. Using CRISPR technology, we created a zebrafish model carrying a DNA lesion similar to that of the affected patient. Homozygous mutant embryos display weak and variable peripheral nerve phenotypes and are viable. Because *ypel3* transcripts are present from fertilization, we generated maternal zygotic mutants. Analysis of these mutants revealed hypomyelination, defective axonal wrapping, and swimming defects. Time-lapse video lineage analysis showed that *ypel3* is required for proper development of oligodendrocytes, Schwann cells, and perineurial cells. These results identify YPEL3 as a new regulator of glial cell development and support the conclusion that the *de novo YPEL3* mutation is responsible for the patient’s disease.

## Introduction

Myelin is a specialized lipid-rich sheath that functions to increase nerve conduction [1–3]. Myelin is essential for neural circuitry function and thus for sensory, motor, and cognitive functions [4]. Within the developing spinal cord, myelinating oligodendrocytes originate from the ventral neural tube [5–9]. Progenitor cells (pMNs) first produce motoneuron progenitors and then switch to generate oligodendrocyte progenitor cells (OPCs) [6,10–12]; those that coexpress Sox10 and Nkx2.2 transcription factors are fated to become myelinating oligodendrocytes [8,13–15]. Myelinating oligodendrocytes migrate along and myelinate developing axons in the central nervous system (CNS) [16,17]. Defects in any of these processes can result in CNS hypomyelination. In the peripheral nervous system (PNS), Schwann cells, which are neural crest derivatives, produce myelin [18,19]. During their migration and interaction with peripheral axons, they express Sox10 [20–22]. Combined expression of Sox10, YAP, and NFATc4 marks the promyelinating stage [21,23,24]. These transcription factors induce expression of Krox20, an essential transcriptional activator that drives peripheral myelination [18]. Therefore, loss of function of common factors involved in the developmental or myelination programs of both oligodendrocytes and Schwann cells, such as Sox10, should impair myelination in both the CNS and PNS.

Perineurial glia are also required for peripheral nerve development [25,26]. They form the perineurium, an epithelial cell layer that ensheaths Schwann cells and peripheral axons, and whose function is similar to that of the blood-brain barrier [27–29]. Perineurial glia associated with motor nerves originate from the ventral domain of the developing spinal cord [26,29,30]. Motor nerve associated perineurial cells leave the CNS at determined exit points, where they migrate in a chain-like fashion along the developing motor nerve [26,29]. During motor nerve morphogenesis, Schwann cells are necessary for proper development of perineurial glia [25,26] and reciprocally, perineural glia are required for proper myelination by Schwann cells, as well as peripheral nerve fasciculation [26,30]. Disruption of instructive interactions between perineural glia and Schwann cells results in peripheral nerve dysmorphogenesis.

We identified a *de novo* heterozygous mutation in the *YPEL3* gene of a patient with CNS hypomyelination and hypertrophic peripheral nerves; this suggested that YPEL3 may function in glia cell development. *YPEL3* was first identified as a senescence factor in a screen for genes induced during the apoptotic phase of myeloid cell development [31]. *YPEL3* belongs to the evolutionarily conserved *Yippee-Like* gene family [32]. Its subcellular localization is highly dynamic during the cell cycle. YPEL3 localizes within the nucleus in a salt and pepper pattern, but is found in an unidentified perinuclear structure during interphase, and near the centrosomes during mitosis [32]. Genetic studies have shown that, under genotoxic stress, *YPEL3* is an inducible target of P53 [33]. In breast, lung, colon, ovarian, and nasopharyngeal carcinoma cells, *YPEL3* expression is downregulated [33–35]. Overexpression of *YPEL3* in cultured cells results in cell cycle arrest [31,33] and suppression of the epithelial-mesenchymal transition in nasopharyngeal carcinoma cells [35]. These data from cell culture models and tumor samples suggest that YPEL3 functions as a tumor suppressor. However, the role of YPEL3 in development remains unknown.

To validate the genetic basis of the patient’s disease and to study YPEL3 function during embryonic development, we created a Ypel3 loss-of-function zebrafish that mimics the patient’s mutation. Our results show that zebrafish depleted of Ypel3 have locomotor defects and that Ypel3 is required for development of myelinating oligodendrocytes, timely exit of the perineurial glia from the developing spinal cord, formation of the perineurium, Schwann cell development, and CNS and PNS myelination. Thus, we propose that Ypel3 is a novel gene that regulates myelinating and perineurial glia development.

## Results

### A *de novo YPEL3* variant is a rare disease-causing candidate allele

**Fig 1:**
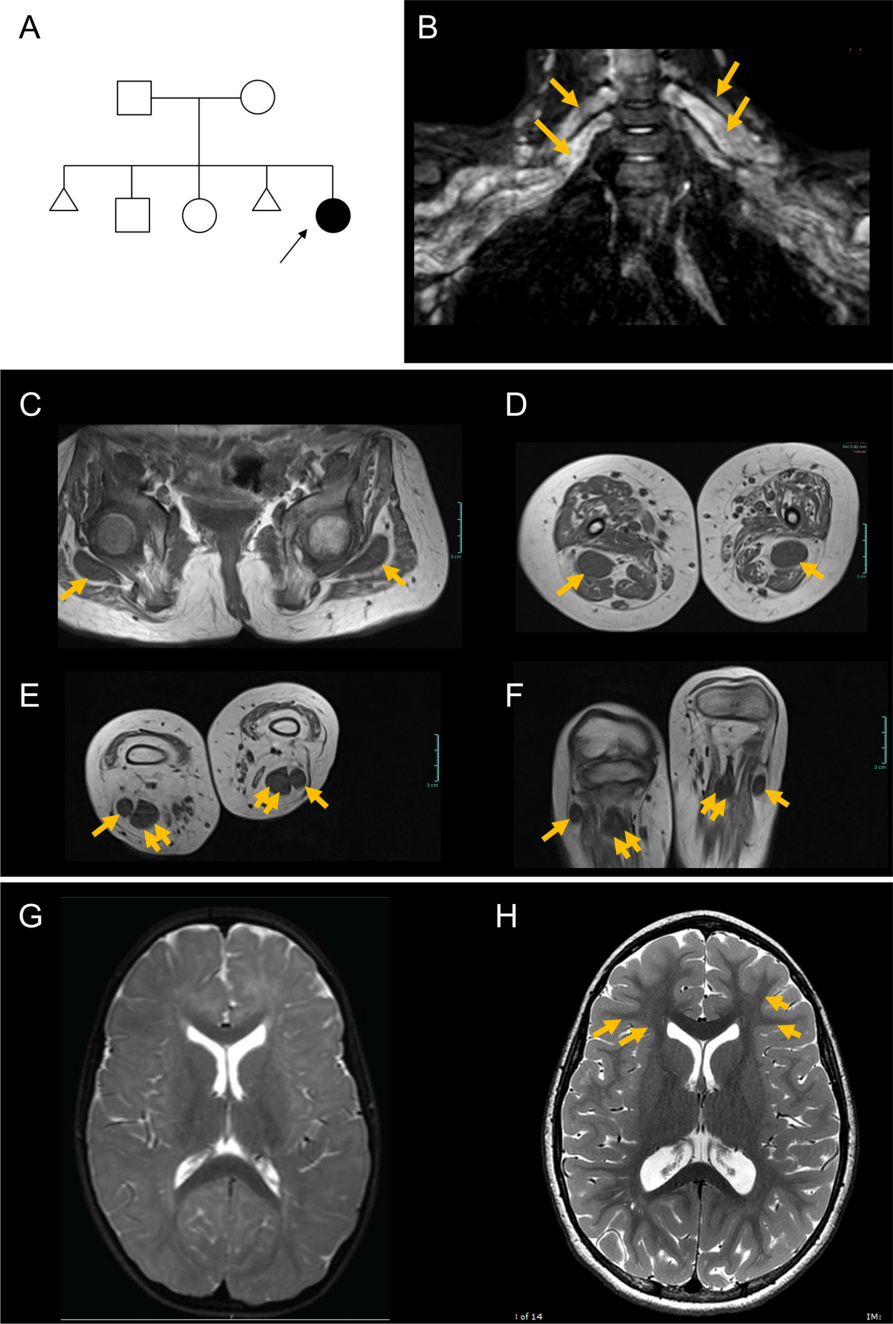
Proband has myelination defects and greatly enlarged nerves. (**A**) Pedigree of the family; arrow points to the proband; triangular shapes indicate instances of spontaneous abortion. (**B-F**) MRI of upper and lower extremities demonstrates enlarged nerves. (**B**) Image showing greatly enlarged brachial plexus. Arrows indicate the thick peripheral nerves. (**C**) Sciatic nerve at the level of the femoral head (arrows). (**D**) Sciatic nerve at the level of the mid-thigh (arrows). (**E**) Posterior tibial (double arrows) and peroneal (single arrows) nerves at their origins. (**F**) Posterior tibial (double arrows) and peroneal (single arrows) nerves at the level of the fibular head. Scale bars, 3 cm. (**G)** Representative brain MRI (axial, T2-weighted) showing poor delineation of white and gray matter, indicating reduced myelination, when compared to age-matched control (**H**). Note the delineation of white and gray matter in control (arrows) that is not seen in the patient.

The patient (UDP_2179) is a Caucasian female who was evaluated at the NIH Undiagnosed Diseases Program at 6 years of age. She was born to a nonconsanguineous union, and has two older siblings who are relatively healthy (**Fig 1A**). However, her mother had two miscarriages. The patient presented with gross and fine motor developmental delay, proximal and distal weakness, lymphedema, profound hypotonia, joint laxity, sleep apnea, and failure to thrive. She had feeding difficulties, with severe gastroesophageal reflux disease (GERD) that required a G-tube placement at 8 months of age. On examination, the patient had normal weight (22.3 kg, 50-75^th^ %ile), height, (120 cm, 25-50^th^ %ile), and body mass index (15.4 kg/m2, 50-75^th^ %ile). She required maximal assistance to come from a supine to a sitting position but could maintain good sitting balance and roll independently from side-to-side. Cranial nerves were normal. Muscle strength was 2–3 (out of 5) in the upper extremities, with no clear strength in the hand intrinsics for grasping, and 0–1 (out of 5) in the lower extremities. She had mild flexion contractures in the hips, knees, ankles, and the interphalangeal joints of both hands. She had intact protective sensation when objectively evaluated with the 5.07 monofilament test, but reported reduced sensation around the hands in a glove-like distribution.

Nerve conduction studies and electromyogram were consistent with a neurogenic process with motor and sensory abnormalities, with significant demyelinating features and axonal loss. Muscle biopsy at 5 years of age showed no primary muscle pathology. MRI of upper and lower extremities showed massive dilation of peripheral nerves throughout the brachial plexus (**Fig 1B**), and in the sciatic (**Fig 1C and 1D**), tibial (**Fig 1E and 1F**) and peroneal (**Fig 1E and 1F**) nerves in the legs, with complete sparing of the cranial nerves and no extension into the spinal cord. The cross sectional diameters of nerves in the arm ranged from 1 to 1.5 cm, with each individual nerve appearing to be composed of a number of engorged fascicles. The caliber of the nerve in the brachial plexus was greater than the diameter of the humerus. There was atrophy throughout the muscles in the arms, but erector muscles in the back appeared to be spared. In the lower extremities, there was marked symmetric diffuse muscle atrophy and fatty infiltration with relative sparing of the adductors. Brain MRI revealed diffuse cerebral hypomyelination comparable to that of a 10 to 12-month-old (**Fig 1G and 1H**).

Quintet exome sequencing failed to yield pathogenic variants in known genes associated with human disease. Reanalysis revealed several bioinformatically interesting candidates (**S1 Table**), including a variant of unknown significance, NM_031477.4(*YPEL3*):c.273dup, that was prioritized and confirmed by Sanger sequencing. This duplication leads to a frameshift, p.Val92Serfs*38, in both long and short YPEL3 transcript isoforms. Clinical genome sequencing did not reveal additional variants.

### *ypel3* is evolutionarily conserved and expressed in the CNS and developing motor nerves

**Fig 2:**
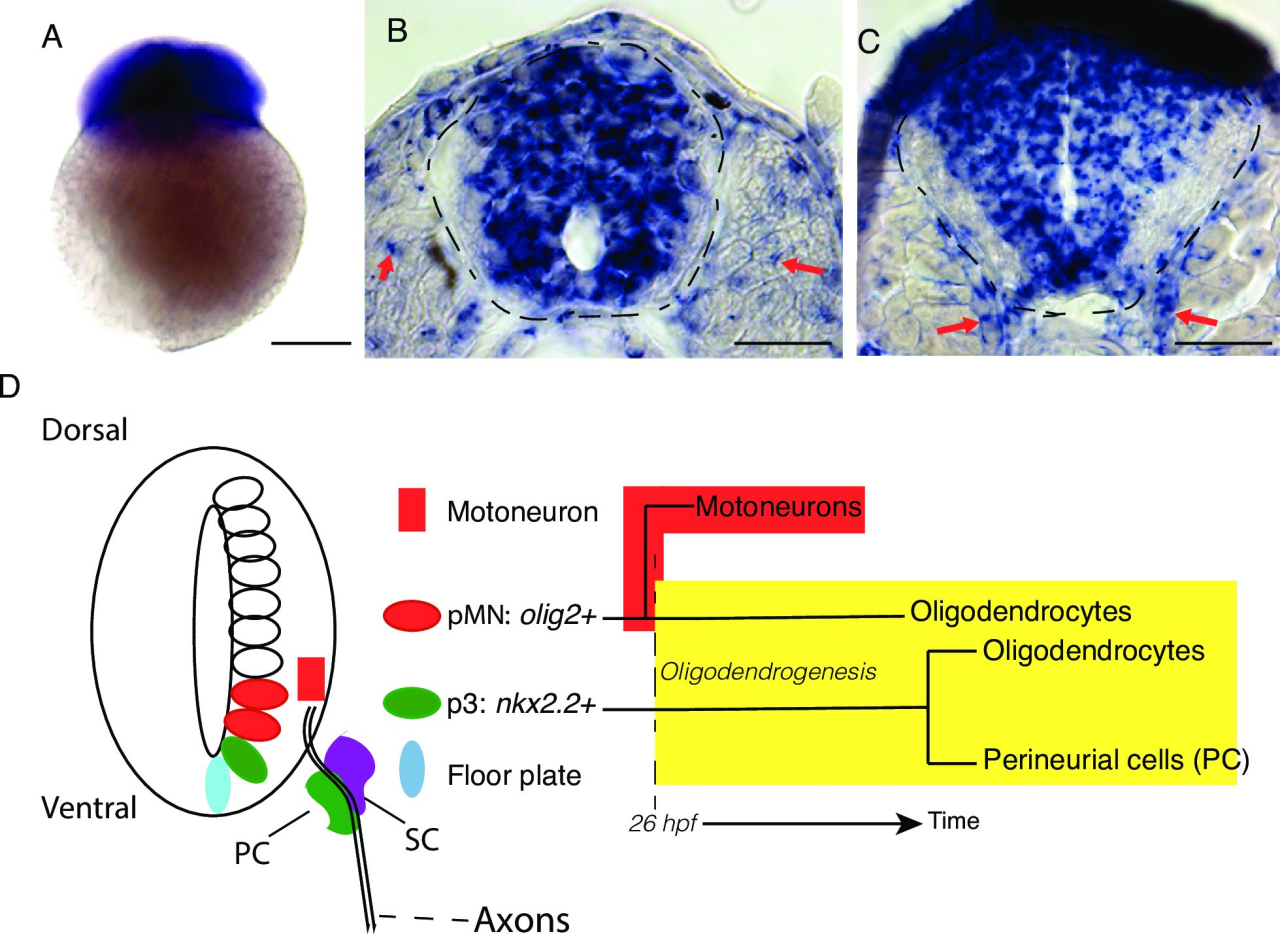
YPEL3 is an evolutionarily conserved disease-causing candidate gene. (**A-C**) *ypel3* is maternally deposited and expressed during neural development in zebrafish. (**A**) Two-cell stage embryo. (**B-C**) Transverse sections. (**B**) At 24 hours postfertilization (hpf), *ypel3* is expressed broadly in the spinal cord and sparsely in the somites (red arrows). (**C**) At 56 hpf, *ypel3* is expressed in the spinal cord and in the region where the motor roots develop (red arrows). **(D**) Diagram of spinal cord domains, their derivatives, and molecular markers. The pMN domain (*olig 2+*) gives rise first to motoneurons, and then later oligodendrocytes (Briscoe et al., 2000; Jessell, 2000; Danesin and Soula, 2017). The p3 domain (*nkx2*.*2+*) is localized ventral to the pMN domain and gives rise to oligodendrocytes and a subset of perineurial cells (Clark et al., 2014; Kucenas et al., 2008b). Schwann cells (SC) are neural crest derivatives. Scale bar: 250 μm in **A**, 25 μm in **B** and **C**.

To investigate the genetic basis of the patient’s disease and YPEL3 function during early development, we used zebrafish. *ypel3*, the zebrafish gene orthologous to human *YPEL3*, encodes a protein that is 89% identical and 94% similar to the human protein (**S1A Fig**). We found that *ypel3* is maternally deposited (**Fig 2A**) and broadly expressed in the developing CNS from 24–80 hours postfertilization (hpf; **Fig 2B and 2C, S1 Fig**). At 24 hpf, *ypel3* expression was also detected in the somite (**Fig 2B**, red arrows). By 56 hpf, expression in the somites was reduced. At this stage and at 80 hpf, *ypel3* mRNA signal delineates the developing motor roots (red arrows in **Fig 2C, S1B Fig**) segmentally arranged at 50–70 μm along the spinal cord, where developing perineurial and Schwann cells are found [25,26,30].

CNS development follows a biphasic mode (**Fig 2D)** [6,36,37]. During the first phase, progenitor domains give rise to distinct arrays of neuronal precursors, including motoneurons. The second phase starts when these progenitor domains switch to generate various types of glial precursor cells [38]. In zebrafish, the pMN produces motoneurons until approximately 26–30 hpf [10]. Then and throughout the second phase, the pMN domain cells gives rise to OPCs, and precursor cells in the p3 domain generate CNS glia and at least a sub-set of perineurial cells [26,30].

### *ypel3*^*b1309*^ and *ypel3*^*b1310*^ CRISPR engineered alleles are equivalent to the *de novo YPEL3* variant

Developmental defects in motoneurons, myelinating oligodendrocytes, Schwann cells and/or perineurial glia could potentially explain the morphological and physiological phenotypes observed in the patient carrying the *de novo* mutation in the *YPEL3* gene. Thus, we engineered *ypel3* CRISPR alleles to study the gene’s role in development.

We generated *ypel3*^*b1309*^ and *ypel3*^*b1310*^ zebrafish mutant alleles using the CRISPR/Cas9 system and a guide RNA targeting the junction between intron 3 and exon 4. The *b1309* allele (p.N56Gfs*1) has a 4 base pair (bp) substitution and a 1 bp insertion, whereas the *b1310* allele (p.G60Rfs*4) carries a 7 bp insertion (**S2A Fig**). As a consequence, the open reading frame is shifted and an early stop codon is introduced by each mutation, resulting in a frameshift similar to that of the patient (p.V54Sfs*38, based on the short *YPEL3* isoform NP_1138996.1) (**S2B and S2C Fig**).

We then characterized motoneuron and glial development in homozygous F2 mutants (see material and methods). Analysis of both *ypel3*^*b1309*^ and *ypel3*^*b1310*^ homozygous mutants revealed rather subtle and variable phenotypes. Considering that maternally deposited *ypel3* products (**Fig 2A**) might provide sufficient function for early development, we generated maternal-zygotic (mz) mutants to remove the maternal contribution. We raised homozygous *ypel3* mutants to adulthood and crossed homozygous mutant females to heterozygous mutant males to generate mz *ypel3* mutants for both alleles. During the first 5 days of embryonic development, there were no gross morphological or behavioral phenotypes in either mz *ypel3* mutant genotype, and mz *ypel3* mutants were viable.

### Specification of the pMN and motoneuron development are normal in the *mz ypel3* mutants

**Fig 3:**
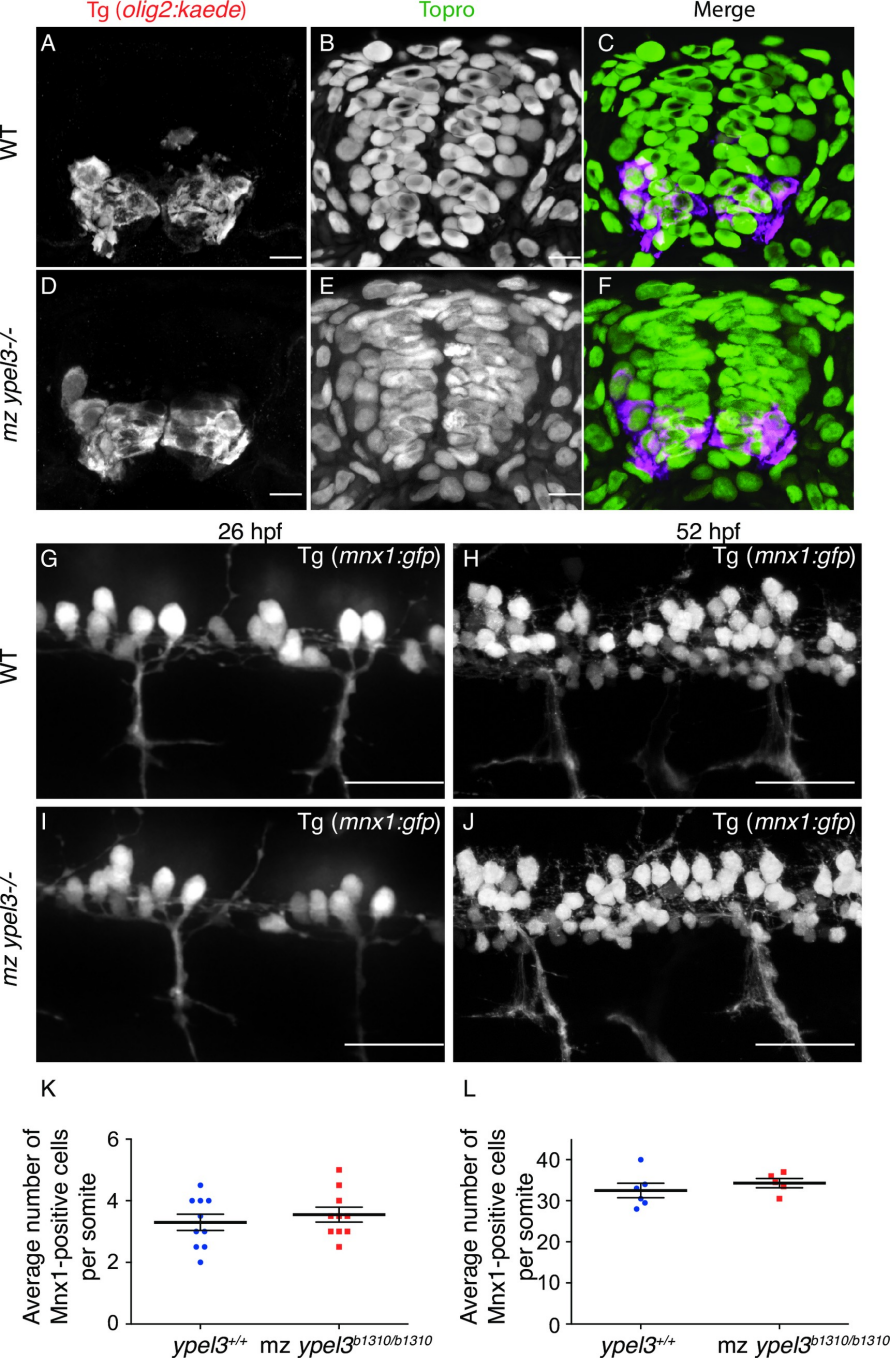
Specification and development of motoneurons are unaffected in maternal-zygotic *ypel3* mutants. (**A-F**) Analysis of the pMN domain and its derivatives. Transverse sections. (**A-C**) WT. (**D-F**) maternal zygotic (mz) *ypel3*^*-/-*^. (**A, D**) pMN domain and its derivatives labeled by *olig2*:*kaede* transgene. (**B, E**) Topro nuclear labeling. (**C, F**) Merge. (**G-J**) Analysis of motoneuron development in mz *ypel3* mutants. Lateral views. Motoneurons are labeled by the *mnx1*:*gfp* transgene. (**G-H**) WT embryo at 26 hpf (**G**) and larva at 52 hpf (**H**). (**I-J**) mz *ypel3* mutant at 26 hpf (**I**) and larva at 52 hpf (**J**). Images were taken at the level of somites 6 to10. (**K-L**) Quantification of the number of motoneurons at 26 hpf (**K**) and at 52 hpf (**L**). Bars show +/- SEM. Scale bars: 5 μm in **A-F**, 25 μm in **G-J**.

To analyze pMN specification, we used the *Tg*[*olig2*:*kaede*] transgenic line, which labels the pMN domain and its derivatives [10,39]. We crossed the transgene into the *ypel3* heterozygous mutant male background. Then, we crossed the transgenic *ypel3* heterozygous males to *ypel3* heterozygous or homozygous mutant females. Wild-type (WT) (n = 10) and transgenic mz *ypel3*^*b1310/b1310*^ mutant (n = 10) embryos were collected at 24 hpf, fixed, individually genotyped, sectioned transversely, and analyzed at the level of somites 6 to 12 (**Fig 3A–3F**). Sections were co-stained with an anti-Kaede antibody and the nuclear maker Topro3 to facilitate cell counting within the pMN domain. In zebrafish at 24 hpf, the pMN is composed of a column 2–3 cells wide adjacent to the luminal region of the developing spinal cord (**Fig 3C and 3F)** [10]. We did not observe any changes in the extent of the pMN along the luminal region of the spinal cord, nor in the number of Kaede-positive cells in mz *ypel3* mutants.

Motoneuron development was assayed by counting cells that express the *Tg*[*mnx1*:*GFP*] transgene, a live marker of differentiated motoneurons [40] that we crossed into the *ypel3*^*b1310*^ heterozygous background. We then crossed these males with *ypel3* heterozygous or homozygous mutant females and used a spinning disk microscope to image 26 hpf embryos and 52 hpf larvae, laterally, at the level of somites 7–10 **(Fig 3G–3J)**. Each imaged embryo or larva was then individually genotyped. Quantification of the GFP-positive cells (**Fig 3K and 3L**) revealed no statistically significant difference between WT and mz *ypel3* mutants at any of the stages studied (n = 10 for each genotype per stage of interest). We also quantified the average motor nerve thickness at 26 and 52 hpf, motor nerve length, and distance between motor nerves at 80 hpf using both *Tg*[*olig2*:*kaede*] and *Tg*[*mnx1*:*GFP*] transgenic lines, and found no difference between WT and mz *ypel3* mutants (**S3 Fig**). Thus, loss of Ypel3 does not affect pMN specification or motoneuron development.

### *ypel3* is required for development of myelinating oligodendrocytes

OPCs coexpressing *nkx2*.*2a* and *sox10* differentiate into myelinating oligodendrocytes [8,41]. Myelinating oligodendrocytes can then be identified by their expression of the *myelin basic protein* (*mbp*) gene [42,43]. Thus, we studied myelinating oligodendrocyte development using dual live colabeling of OPCs using the *Tg*[*sox10*:*mRFP*] and *Tg*[*nkx2*.*2a*:*GFP*] transgenes [8,17], referred as *sox10*:*mRFP* and *nkx*.*2*.*a*:*GFP*, respectively, here after. As expected, the *nkx2*.*2a*:*GFP* transgene was also detected in the ventral p3 domain of the spinal cord [8]. In our experiments, *ypel3*^*b1310*^ heterozygous males carried the *nkx2*.*2a*:*GFP* transgene, whereas the *sox10*:*mRFP* construct was carried by either *ypel3* ^*b1310*^ heterozygous or homozygous mutant females.

**Fig 4:**
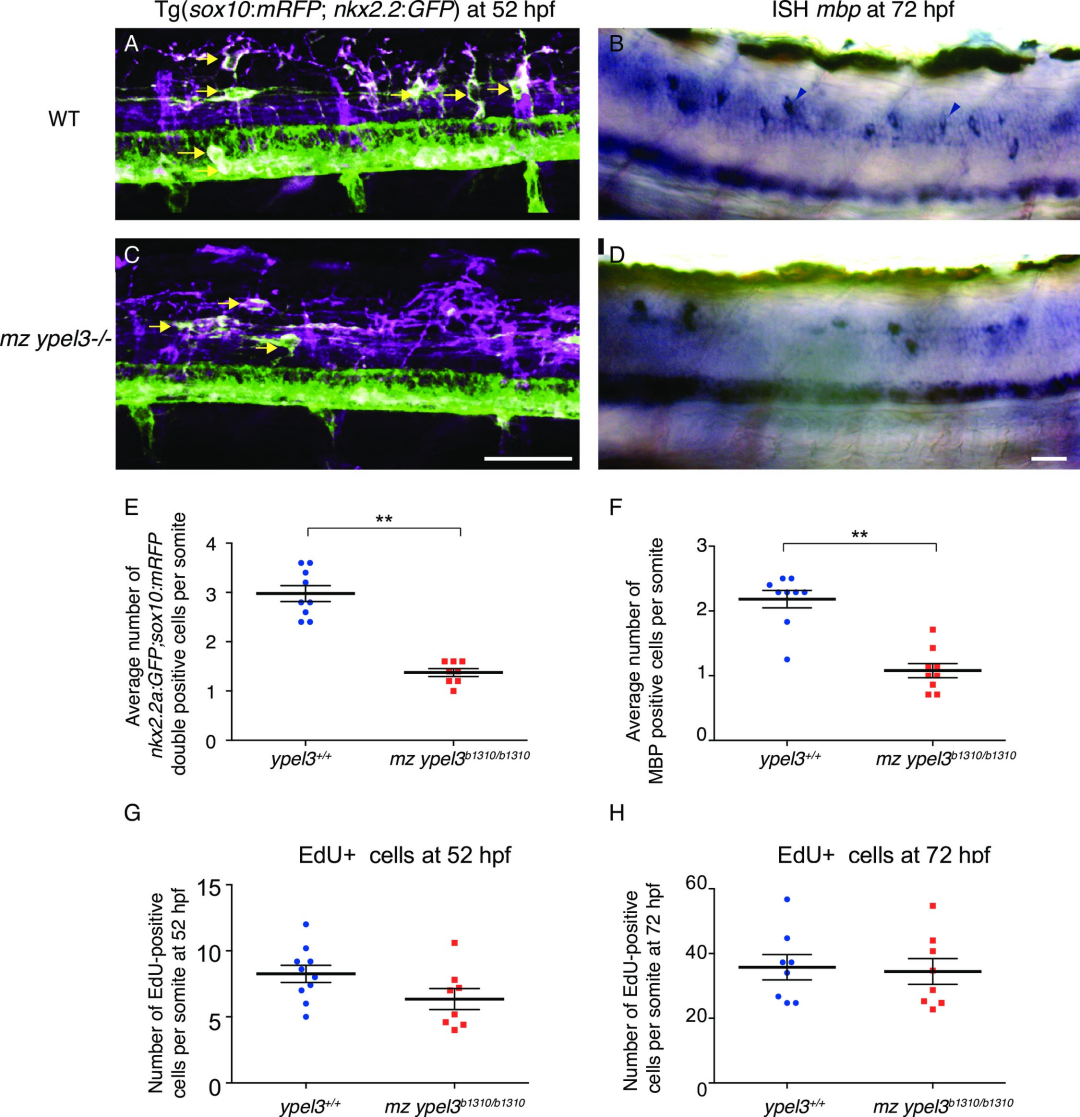
*ypel3* is required for myelinating oligodendrocyte development. (**A-B**) WT larvae at 52 hpf (**A**) and at 72 hpf (**B**). (**C-D**) mz *ypel3* mutants at 52 hpf (**C**) and at 72 hpf (**D**). Arrows indicate myelinating oligodendrocytes (double mRFP, mGFP positive cells) in (**A**) and (**C**). Black arrowheads show myelinating oligodendrocytes expressing *mbpb* in (**B** and **D**). Scale bars: 25 μm. Images are lateral views. (**E-H**) Quantification of the number of myelinating oligodendrocytes at 52 hpf (**E**), *mbpb* expressing cells at 72 hpf (**F**), EdU positive cells at 52 hpf (**G**) and 72 hpf (**H**). Bars represent +/- SEM.

After crossing the transgenic *ypel3* heterozygous males with transgenic *ypel3* heterozygous or homozygous females, we fixed double GFP-positive (GFP+) and mRFP-positive (mRFP+) larvae at 56 hpf, and genotyped each larva. WT (n = 9) and mz *ypel3* (n = 10) mutant larvae were labeled with antibodies recognizing GFP and mRFP. The average number of GFP and mRFP double positive cell bodies localized dorsal to the p3 domain were quantified per somite between somites 6 and 12 (**Fig 4A and 4C**). We found a statistically significant 50% decrease in the number of GFP and mRFP double positive oligodendrocytes in the mz *ypel3* mutants compared to WT. At this stage, in WT larvae, the average number of GFP and mRFP double positive oligodendrocytes was 3 per somite, whereas in mz *ypel3* mutants this number ranged from 1 to 2 (**Fig 4E**).

To understand how the dynamics of oligodendrocyte development is affected in *ypel3* mutants, we performed live imaging with a spinning disc microscope. For this, we mounted mRFP+, GFP+ larvae laterally and recorded oligodendrocyte development from 50 hpf to 62 hpf at the level of somites 8–10, followed by genotyping. Analysis of the time-lapse videos revealed that, from 50 hpf, the number of myelinating oligodendrocytes was constant in WT larvae (n = 3), suggesting that no new cell bodies within the region of interest (localized dorsal of the p3 domain) were added (**S1 Video**). However, in the mz *ypel3* mutants, the number of myelinating oligodendrocytes was strongly reduced at 50 hpf (n = 5; **S2 video**). At this stage, mz *ypel3* mutants have almost no myelinating oligodendrocytes (**S4A Fig**). From 52 hpf in mz *ypel3* mutants, myelinating oligodendrocytes gradually emanate from the ventral p3 domain and migrate dorsally, increasing the number of presumptive myelinating mutant oligodendrocytes to levels similar to those of WT **(S4A Fig**). Despite the apparent recovery, the mz *ypel3* myelinating oligodendrocytes showed morphological defects compared to WT including a reduced number and length of filopodia. Because filopodia are dynamic structures, we imaged cells at various time points (50, 52, 54, 56, and 58 hpf). The average number of filopodia per myelinating oligodendrocyte in WT during this period was 3, whereas myelinating oligodendrocytes in mz *ypel3* mutants formed only 2 on average (**S4B Fig**, n = 68 cells for both WT and mutants, p< 0.0001). In addition, the average WT filopodial length (**S4C Fig**) was 8 μm (n = 116 filopodia) whereas mutant filipodia were only 5 μm long on average (n = 133 filopodia). Thus, loss of Ypel3 function causes a delay in the production of myelinating oligodendrocytes and disrupts development of oligodendrocyte filopodia.

Filopodia number and extension are necessary for correct axonal wrapping and myelination [17,43,44]. We assayed myelin expression by oligodendrocytes at 72 hpf by *in situ* hybridization (ISH; **Fig 4B and 4D**), and observed a 50% decrease in the number of *mbpb-*positive cells in mz *ypel3* mutants compared to WT (**Fig 4F**). We counted the number of *mbpb*-positive cells in the spinal cord region localized between somites 6 to 10. In WT, the *mbpb*-positive cells were segmentally arranged in a uniform pattern **(Fig 4B**, arrowheads); each somite-long segment contained an average of two *mbpb*-positive cells **(Fig 4F**). In mz *ypel3* mutants, however, each somite segment had an average of one *mbpb-*positive cell **(Fig 4F**). Mutant *mbpb*-positive cells were discontinuously distributed along the spinal cord and segments were often devoid of *mbpb-*positive cells **(Fig 4D)**.

To determine whether the decrease in the number of *mbpb-positive* cells was due to cell death or a defect in cell proliferation, we performed TUNEL (terminal deoxynucleotidyl transferase dUTP nick end) and EDU (5-ethynyl-2’-deoxyuridine) labeling, respectively. TUNEL labeling was performed in 32, 56 and 72 hpf WT and *mz ypel3* mutants. Larvae were analyzed at the level of the spinal cord between somites 6 to 12. At all stages, the number of TUNEL-positive cells was zero for both WT and mutants (n = 45 for each stage and genotype). We observed TUNEL signal in the heart at these stages, as a positive control. These experiments ruled out cell death as a cause for the decrease in the number of *mbp*-positive cells in mz *ypel3* mutants.

We then performed EDU pulse and chase experiments to study cell proliferation. The “pulse” group was fixed at 52 hpf and the “chase” group was fixed at 72 hpf. There was no statistically significant difference in the average number of EDU-positive cells per larva at 52 and 72 hpf between WT and *mz ypel3* mutants **(Fig 4 and 4H),** however there was no apparent localization of EDU within the oligodendrocyte population. We then used the Tg[*olig2*:*kaede*] transgenic line, which labels pMN derivatives including motoneurons and oligodendrocytes to examine proliferation of these cell types. We reasoned that proliferation changes in the oligodendrocyte population could be revealed by photoconverting Kaede early and examining the larvae later; newly born cells would be labeled green, and older photoconverted cells red. We crossed Tg[*olig2*:*kaede*] heterozygous *ypel3* males with Tg(*sox10*:*mRFP*) homozygous or heterozygous *ypel3* mutant females. Photoconversion was done at 26 hpf, and we counted double green Kaede-positive (Kaede+) and mRFP+ oligodendrocytes at 80 hpf. We did not find a difference in the average number of green Kaede+, mRFP+ double labeled oligodendrocytes (**S5A–S5C Fig**). In addition, the number of myelinating oligodendrocytes was the same in WT and mz *ypel3* mutants at 7 days postfertilization (dpf) (**S5D Fig**). These experiments showed that *ypel3* loss of function transiently affects the myelinating oligodendrocyte population and its development during CNS development.

### Ypel3 is required for proper development of Schwann cell precursors

Enlarged peripheral nerves are one of the most striking phenotypes of the patient (**Fig 1B and 1F**). Taking advantage of the *Tg*(*olig2*:*kaede)* and *Tg*(*mnx1*:*GFP*) transgenic lines that label motoneuron cell bodies and axons, we found no differences in the width of axon bundles at 24 and 52 hpf between mz *ypel3* mutants and WT siblings (**S3A and S3B Fig**). This suggested that the hypertrophic nerve defect does not have a neuronal origin.

Expression of *ypel3* mRNA was detected in cells associated with the motor roots (**Fig 2C**). Therefore, we examined development of peripheral glia to determine whether *ypel3* is required for peripheral motor nerve morphogenesis. Schwann and perineurial cells are the two types of glial cells required for peripheral motor axon myelination and fasciculation [29]. We labeled Schwann cells with the *sox10*:*mRF*P transgene and perineurial cells with the *nkx2*.*2*:*GFP* transgene. Time lapse recording of mz *ypel3* mutants from 50 to 60 hpf revealed that perineurial glia (green arrows in **S1 Video**) did not exit the CNS in a timely manner and failed to wrap the developing motor nerves (compare **S1 and S2 Videos**).

Motor axon ensheathing can be characterized as a 3-step process. First, Schwann cell precursors (SCP) migrate ventrally and encounter the developing axons. During this step, the SCPs differentiate into immature Schwann cells and associate with motor axons, forming tube-like structures. In zebrafish, the second step starts around 40–48 hpf [26], when perineurial glia leave the CNS and migrate along the immature motor nerve. Perineurial glia envelop the immature Schwann cells, becoming part of the tubular structure. During the third step of “tightening” or “compaction”, both glial cell types become tightly associated with each other and perineurial cells ensheath the axons and associated Schwann cells, forming the perineurium. This tightening process can be visualized by studying the formation of slender rods that emanate from the ventral roots, and the co-expression of markers labeling both Schwann and perineurial cells.

**Fig 5:**
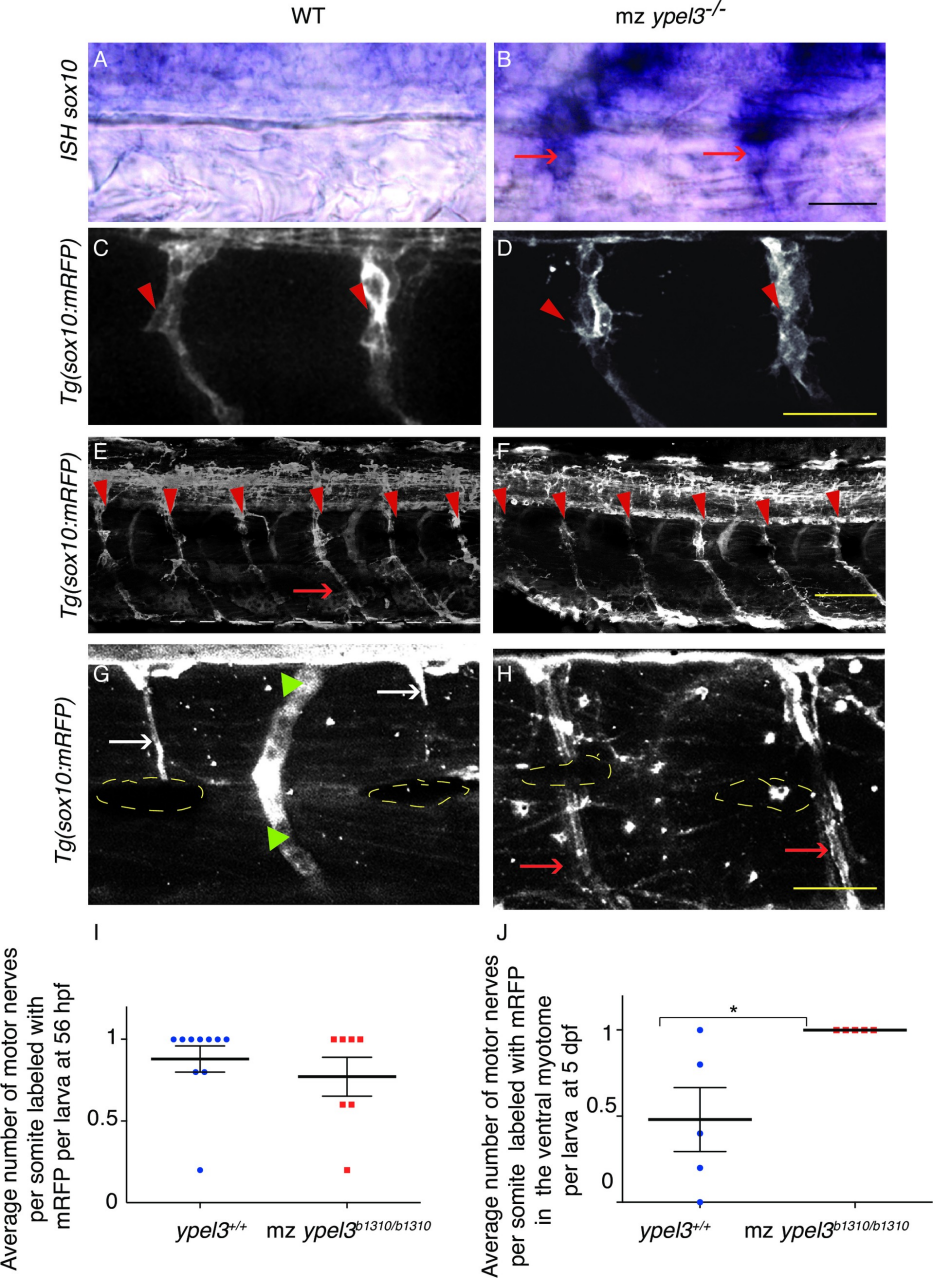
Ypel3 is required for proper development of Schwann cells. (**A-B**) *sox10* mRNA *in situ* hybridization (ISH) in WT (**A**) and mz *ypel3* mutant (**B**) showing maintained *sox10* expression (red arrows) in the mz *ypel3* mutant at 26 hpf. (**C-D**) Schwann cell precursors migrate correctly in the region of the developing motor nerves in the mz *ypel3* mutant. WT (**C**) and mz *ypel3* mutant (**D**) embryos at 26 hpf. Arrowheads indicate Schwann cell precursors. (**E-F**) Initial ensheathing of motor axons by Schwann cells occurs properly in the mz *ypel3* mutant. WT (**E**) and mz *ypel3* mutant (**F**) larvae at 56 hpf. Arrowheads indicate motor nerves. Arrow indicates ventral Schwann cells ensheathing motor nerve. (**G-H**) Schwann cells fail to form normal structures in the mz *ypel3* mutant. At 5 days postfertilization (dpf), in WT (**G**), Schwann cells form slender rod-like structures (arrows). At this stage, the mRFP signal has cleared from the ventral myotome. In the mz *ypel3* mutant (**F**), Schwann cells fail to wrap the motor axon tightly and *sox10* expression as indicated by mRFP is maintained within the ventral region of the myotome. Yellow dashed lines outline melanocytes. Green arrowheads indicate blood vessels. All images are lateral views. Scale bars: 25 μm. (**I**) Quantification of the average number of motor nerves per somite labeled with *sox10*:*mRFP* at 56 hpf. (**J**) Quantification of the average number of motor nerves per somite labeled with *sox10*:*mRFP* at the level of ventral myotome. Bars represent +/-SEM.

In zebrafish, SCPs and immature Schwann cells are labeled by the *sox10*:*mRFP* transgene and Schwann cell maturation correlates with the progressive fading of transgene expression in a ventral to dorsal gradient along the motor nerve [26]. Thus, we characterized initial Schwann cell development by assaying the expression of *sox10* at 26 hpf using colorimetric whole-mount *ISH* and Nomarski microscopy. Surprisingly, *sox10* expression levels were elevated in the mz *ypel3* mutant Schwann cells compared to WT (**Fig 5A and 5B**; n = 15 for each experiment). When we assayed Schwann cell localization at this stage quantifying labeled mRFP+ cells along the motor nerve, we did not observe any difference in the number of cells (**Fig 5C and 5D; S6 Fig**). This suggested that high levels of *sox10* expression did not affect SCP migration.

At 56 hpf, during the early steps of the second phase of motor axon ensheathing, Schwann cell localization and morphology along the motor nerve were indistinguishable between mz *ypel3* mutant and WT (**Fig 5E and 5F)**. In addition, scoring mRFP-positive cells along the motor nerve, revealed that essentially all motor nerves were associated with Schwann cells (**Fig 5I**) in both WT and *mz ypel3* mutants.

At 5 dpf, during the late stages of the third phase of motor axon ensheathing, WT Schwann cells have acquired the typical slender rod morphology, and *sox10*:*mRFP* expression has faded from the ventral myotome region (**Fig 5G**, white arrows). In the mz *ypel3* mutants, however, 100% of the Schwann cells failed to acquire the slender rod morphology, but rather appeared bloated and only loosely associated with the nerve, and *sox10*:*mRFP* expression was maintained in the ventral myotome (**Fig 5H**, red arrows; **Fig 5J**). This suggested a defect in Schwann cell development in the mz *ypel3* mutant.

### Ypel3 is required for perineurial glia development

Perineurial glial cells derive from the ventral region of the developing spinal cord. This domain and its cell derivatives are labeled by the *nkx2*.*2*:*mGFP* transgene. In zebrafish, perineurial cells start exiting the CNS by 40 hpf and migrate along the motor nerve to continue their development and formation of the perineurium [26].

**Fig 6:**
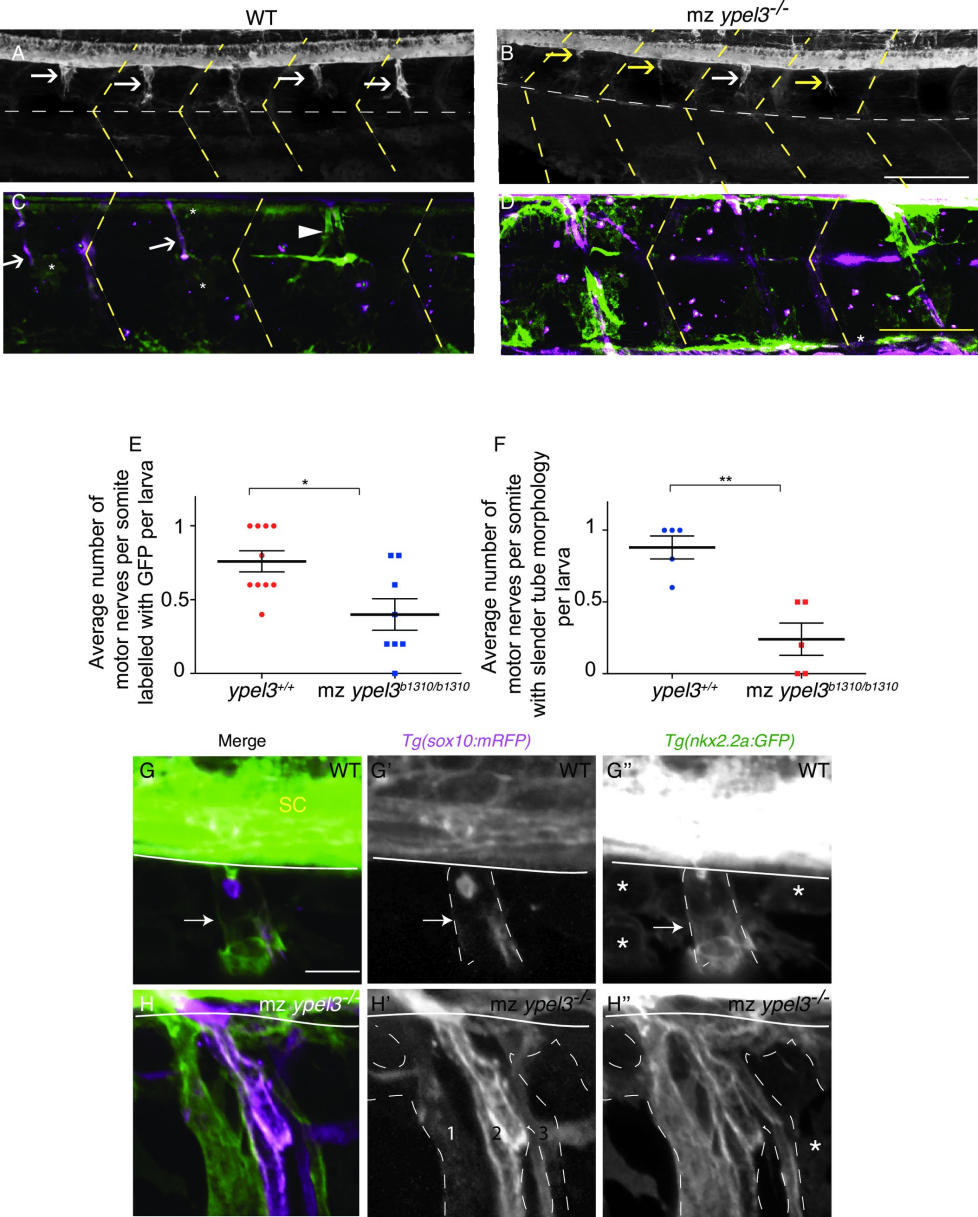
Ypel3 is required for perineurial glia ensheathing. (**A**) WT larva at 56 hpf. Perineurial glia (expressing *nkx2*.*2a*:*GFP*, green) have migrated out of the CNS and begun to ensheath the motor nerve. White arrows indicate perineurial glia associated with the immature motor nerve. (**B**) mz *ypel3*^*-/-*^ mutant at 56 hpf. At this stage, perineurial glia are largely absent, and the few that migrate out of the CNS appear wispy and thin (yellow arrows). (**C**) In WT larvae at 5 dpf, perineurial glia are tightly associated with Schwann cells (expressing *sox10*:*mRFP*, magenta) forming slender tubes (white arrows). A small percentage of WT perineurial cells have not formed these slender tubes (arrowhead). (**D**) In mz *ypel3*^*-/-*^ mutants, at 5 dpf, perineurial glia are overgrown and fail to associate with the Schwann cells. Yellow dashed lines label the somite boundaries. Scale bar: 25 μm. (**E-F**) Quantification of the average number of motor nerves per somite labeled with GFP (**E**) and the average number of motor nerves per somite with slender tube morphology (**F**). (**G-G”**) WT larvae at 7 dpf. Perineurial glia have completely wrapped the Schwann cells forming a tubular structure (arrows). Some mRFP expression remains within the Schwann cells. (**G**) Merge. (**G’**) mRFP expression is clustered within the GFP positive domain (outlined with white dashed lines), which is composed of perineurial cells. (**G”**) Perineurial cells form a tubular structure (outlined with white dashed lines). (**H-H”**) mz *ypel3* mutant at 7 dpf. Perineurial glia are overgrown and form a defasciculated structure. (**H**) Merge. (**H’**) mRFP signal is elevated in the mz *ypel3* mutant Schwann cells and delineates three defasciculated structures (1, 2, 3). (**H”**) mz *ypel3* mutant perineurial glia fail to wrap Schwann cells correctly resulting in an enlarged nerve (outlined with white dashed lines). White horizontal line in (**G-H”**) spinal cord ventral border. White horizontal dashed line (**A-D**) horizontal myoseptum. SC: spinal cord. *: loose perineurial cells. All images are lateral views. Scale bars: 25 μm in A-D, 5 μm in G-H”.

We used time-lapse videos from 50 to 60 hpf to track GFP-positive perineurial cells exiting the spinal cord and found that perineurial glia failed to migrate properly along the immature motor nerves in mz *ypel3* mutants. In WT, this migratory process is highly dynamic, as perineurial cells actively extend and retract cellular processes along their migratory track, contacting and wrapping Schwann cells, and finally forming a tubular structure (**S1 Video**). In mz *ypel3* mutants, very few perineurial glia were observed. A few filopodia sometimes emanated from the spinal cord, but no cell bodies (**S2 Video**). To quantify defects, we scored the presence of GFP-positive perineurial cells associated with mRFP+ Schwann cells along the motor axons. WT and mz *ypel3* mutant larvae were fixed at 56 hpf, a relatively well-advanced stage for the wrapping of the immature nerve by the perineurial cells, and antibody labeling for both mGFP and mRFP was performed. 80% of WT motor nerves were associated with perineurial glia (**Fig 6A and 6E**), and perineurial glia formed easily distinguishable tubular structures that enveloped the base of the motor axons and Schwann cells from the spinal cord to the horizontal myoseptum (**Fig 6A,** white arrow and dashed white line, respectively). In *mz ypel3* mutants, on the other hand, the percentage of motor nerves associated with perineurial glia was 40%, a reduction of 50% in comparison to WT (**Fig 6B and 6E**). In addition, the few mutant perineurial cells that migrated out from the CNS failed to form rod-like structures, indicative of a defect in wrapping (**Fig 6D and 6F**).

To learn whether these defects were due simply to a delay in development, we grew the larvae to 5 dpf and labeled them as described above. As expected, we observed slender rods colabeled with mRFP and GFP in WT (**Fig 6C**, white arrows), showing that the tightening phase is being executed properly. A few loose perineurial glia were observed around the slender rods (**Fig 6C**, arrowhead). However, in *mz ypel3* mutants, we observed overgrown perineurial glia, as indicated by expression of the *nkx2*.*2*:*mGFP* transgene, that formed misshapened and swollen tubular structures. These large nerves (**Fig 6D**) were very reminiscent of the hypertrophic nerves observed in the patient (**Fig 1B–1F**). By 5 dpf in WT siblings, 90% of the peripheral glia associated with motor nerves (n = 22) had acquired a slender rod morphology (**Fig 6C and 6F**), but this percentage was only 25% in the mz *ypel3* mutants (n = 22) (**Fig 6D and 6F**). Structurally, WT slender rods at 7 dpf were composed of a tubular perineurial cell layer that encapsulated the Schwann cells (**Fig 6G–6G”**, arrows), which progressively extinguished *sox10*:*mRFP* transgene expression. In contrast, perineurial cells in mz *ypel3* mutants were very disorganized and failed to form a slender tubule. Instead, they gave rise to a swollen partitioned perineurium, and the motor nerve was defasciculated as visualized with mRPF expression in Schwann cells (**Fig 6H–6H”**), reminiscent of the hypertrophic nerves of the patient. These results indicate that mutant perineurial glia do not develop properly and fail to ensheath Schwann cells and motor axons, hence compromising the structural integrity of the perineurium and contributing to nerve defasciculation.

### mz *ypel3* mutants have elevated levels of phosphatidic acids and very long-chained ceramides and lower levels of Myelin basic protein-B and galactolipids

**Fig 7:**
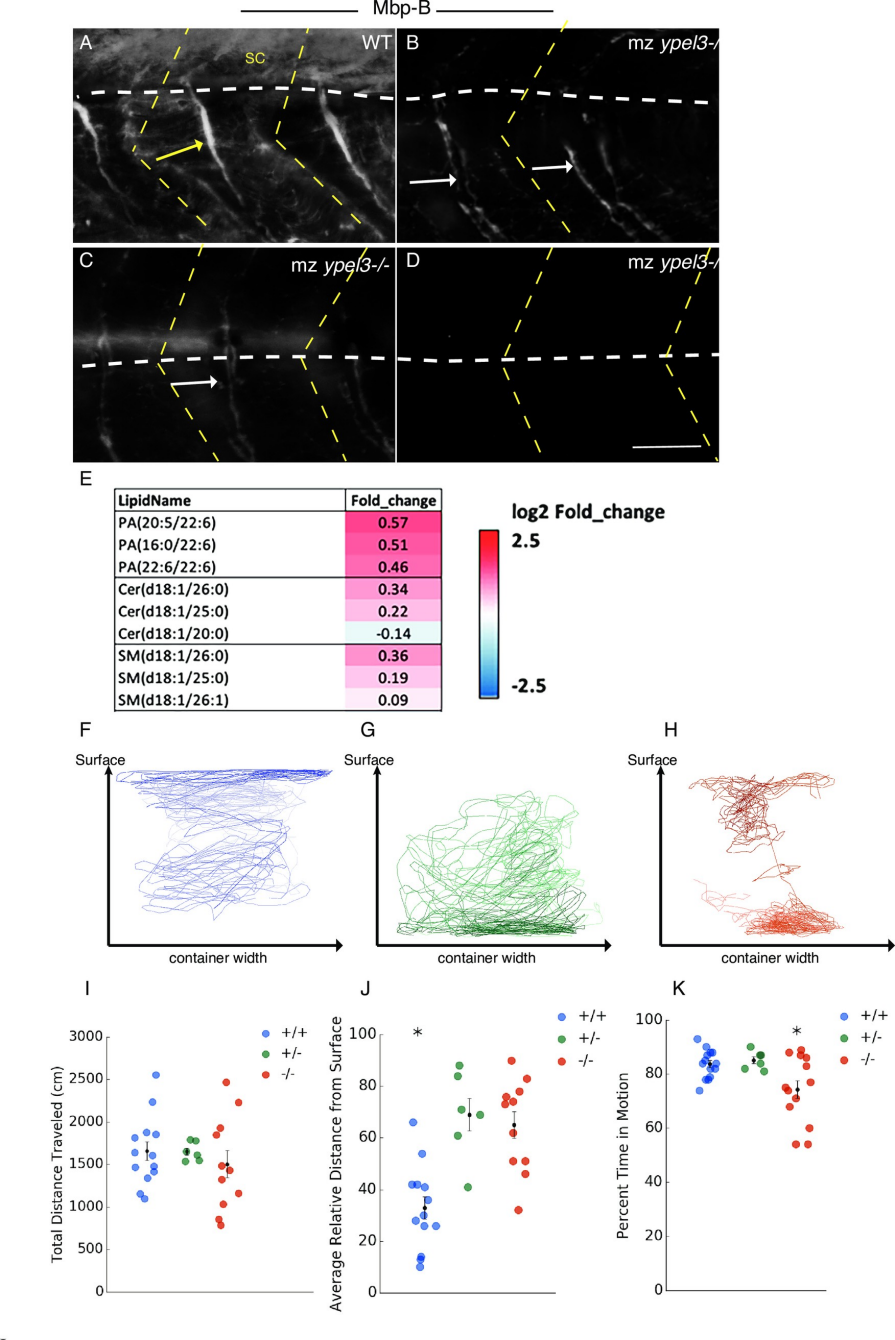
Ypel3 is required for myelination and locomotory behavior. (**A-D**) Myelin basic protein b (Mbpb) expression. Lateral views of embryos labeled for Mbpb at 7 dpf. (**A**) WT. (**B-D**) mz *ypel3* mutants. Mbpb levels are undetectable in the spinal cord (sc) and variably reduced in the motor nerves (arrows). Yellow arrow shows a fasciculated nerve with a homogenous distribution of the Mbpb signal in WT. White arrows show defasciculated nerves in mz *ypel3* mutants. Mutant nerves have variable levels and an uneven distribution of Mbpb. White horizontal dashed lines indicate the spinal cord ventral border in (**A-B**) and the horizontal myoseptum in (**C-D**). Yellow dashed chevron: somite borders. Scale bar: 25 μm. (**E**) Lipidomics panel showing an increase in phosphatic acids (PA) and ceramides (Cer) except Cer(d18:1/20:0) in the mz *ypel3* mutant compared to WT. (**F-K**) Adult mz *ypel3*^*-/-*^ mutants have behavioral and locomotion defects. (**F-H**) Representative swimming traces of WT (**F**), heterozygous *ypel3*^*b1309*^ (**G**), and homozygous mz *ypel3*^*b1309*^ mutants (**H**). (**I-K**) Quantification of total distance traveled (**I**), average relative distance from the surface (**J**), and percent time spent in motion (**K**). +/+: WT. +/-: heterozygous. -/-: mz *ypel3* mutants. Bars represent +/- standard error of the mean (+/- SEM).

We further examined myelinating oligodendrocyte and Schwann cell development in 7 dpf larvae by whole-mount immunolabeling of Myelin basic protein-b (Mbp-b), a marker of this late developmental step. We detected Mbp-b expression within the CNS and along each motor nerve in 100% of WT larvae (n = 12) (**Fig 7A**). In mz *ypel3* mutants, Mbp-b was undetectable in the CNS of 100% of larvae (n = 14). Moreover, Mbp-b signal was discontinuous in mz *ypel3* mutant motor nerves and weaker than in WT in 79% of the cases (10/14). Mbp-b signal often highlighted defasciculated nerves in these mutants (**Fig 7B and 7C**, arrows). In the remaining 21% (3/14), Mbp-b was undetectable in the motor nerves (**Fig 7D**).

To characterize biochemical phenotypes of the mutant Schwann and perineurial cells that could potentially play a role in their failure to differentiate, we performed comparative lipidomics analysis of WT and mz *ypel3* mutant zebrafish larvae at 7 dpf. For this, 5 independent replicates of 400–500 larvae each were collected at 7 dpf, frozen in liquid nitrogen and processed for lipidomics analysis. The mz *ypel3* mutants had altered levels of sphingomeylin, ceramides, and phosphatidic acids compared to WT (**Fig 7E)**. Sphingomyelins, major lipid components of myelin membranes, are decreased in *ypel3* except for those containing a fatty acid chain of 24 carbons or greater. The ceramide species that are statistically elevated also contain longer chain fatty acids (C25 and C26). Fatty acid chain length has been linked to neuronal cell death [45,46]. Previous studies in the CNS have shown that galoctosphingolipids, ceramide derivatives that are decreased in mz *ypel3* mutants (**Fig 7E**), act as regulators of oligodendrocyte development [47]. Phosphatidic acids were elevated in mz *ypel3* mutants, and previous studies in the PNS showed that high levels of phosphatidic acids negatively regulate Schwann cell development and myelination through the activation of the MEK-ERK pathway [47]. Thus, defects in OPC and Schwann cell development in mz *ypel3* mutants may be due to changes in lipid composition. Together, these results strongly suggest that Ypel3 function is required for myelinating oligodendrocyte and motor nerve development.

### Maternal zygotic *ypel3* mutants have behavioral and locomotor defects

To understand the functional consequences of these developmental defects, we raised mz *ypel3* mutants to adulthood and analyzed their swimming behavior. We measured the total distance the mz mutant fish traveled, preference for tank space occupancy, and time in motion [48] compared to WT and heterozygous siblings (**Fig 7F–7K**). The total distance traveled did not differ among the different genotypes (p > 0.950 in all cases; **Fig 7I**). In contrast, mz *ypel3* and heterozygous mutant fish exhibited a significant preference for the bottom of the tank, as measured by average relative distance from the surface of the water compared to WT siblings (p < 0.001 in both cases), and were similar to each other (p = 0.832) (**Fig 7F–7H and 7J**). Strikingly, mz *ypel3* mutants spent less time in motion than either heterozygous or WT siblings (p = 0.020 and 0.011, respectively), whereas behavior of heterozygotes and WT did not differ (p = 0.946; **Fig 7K**). This difference was due to more frequent pauses by the mz *ypel3* mutants during their swimming, compared to siblings (heterozygotes, p = 0.032; WT, p = 0.013). The reduced motility of mz and heterozygous *ypel3* mutants was reminiscent of the reduced motor skills of the heterozygous patient.

## Discussion

The developmental role of Ypel3 was previously unknown. Our studies demonstrate that *ypel3* is required for development of myelinating oligodendrocytes and motor nerve morphogenesis. Despite broad expression of *ypel3* in the developing spinal cord, we find that neither pMN specification nor motoneuron differentiation is affected in *ypel3* mutants. Similarly, there is no sign of increased cell death or decreased cell proliferation. On the other hand, onset of the earliest known markers for the myelinating OPC lineage (*sox10* and *nkx2*.*2*) is delayed, and later development of OPCs is defective. Lipidomics and molecular analysis using Mbp-b as a marker show that the few specified myelinating oligodendrocytes fail to differentiate properly. Thus, our data suggest that Ypel3 functions for proper development of perineurial and myelinating glial cells.

Temporal delay in the appearance of myelinating oligodendrocytes in the *mz ypel3* mutants, as observed with time-lapse microscopy, suggests that Ypel3 influences the timely onset of the transcriptional cascade regulated by Sox10 and Nkx2.2 transcription factors. Delay in the onset of this cascade results in a decrease in the total number of myelinating oligodendrocytes, as in *Nkx2*.*2* mouse mutants [41]. In addition, the few presumptive mz *ypel3* mutant myelinating oligodendrocytes that do form are discontinuously distributed along the developing spinal cord and have abnormal morphologies characterized by fewer and shorter filopodia. These defects, the decrease in the population of *mbpb+* oligodendrocytes, their uneven distribution, and abnormal filopodia, likely contribute to the hypomyelination phenotype [17,43,49–52] we observed in the mz *ypel3* mutants. Future studies to understand how Ypel3 promotes myelinating oligodendrocyte development will need to identify Ypel3 interactors and the downstream biochemical pathways affected in the mutant, as well as elucidating the cellular functions of Ypel3, which remain unknown.

An important driving force during peripheral nerve morphogenesis is the establishment of instructive interactions between perineurial glia and Schwann cells [25,26,30]. Disruption of these interactions causes defasciculation and hampers myelination by Schwann cells. During motor nerve development, both perineurial and Schwann cells are affected in mz *ypel3* mutants. Although high levels of *sox10* expression are maintained, Schwann cells still migrate toward the motor axons. Similarly, initial wrapping of the motor nerve by Schwann cells occurs normally. However by 5 to 7 dpf, wrapping defects are evident when Schwann cells fail to form slender rods, stop expressing the immature Schwann cell transgene marker *sox10*:*mRFP*, and fail terminal differentiation as indicated by Mbp-b expression. Further, our lipidomics analysis revealed elevated levels of phosphatidic acids in mz *ypel3* mutants compared to WT, a dysregulation associated with impaired Schwann cell development [53]. These late stage defects in Schwann cell development correlate with the time that perineurial glia form the perineurium. Our data also indicate that *ypel3* is necessary much earlier for the timely migration of perineurial glia and their association with Schwann cells. We propose that initial failure of perineurial glia migration disrupts the instructive interaction between Schwann cells and perineurial glia. As a consequence, Schwann cells cannot differentiate properly and Mbp-b levels are decreased in mz *ypel3* mutant motor nerves. In support of this interpretation, in both mouse and zebrafish models depleted of perineurial cells, Schwann cells fail to differentiate properly and nerves have severe myelination and fasciculation defects, [26,30], as we observed in mz *ypel3* mutants.

YPEL3 has been proposed to act as a negative regulator of the WNT/ß-Catenin signaling pathway by preventing ß -Catenin nuclear translocation during metastasis of nasopharyngeal carcinoma cells [35]. According to this model, YPEL3 blocks the epithelial-mesenchymal transition by stabilizing cytoplasmic ß-Catenin levels, although the regulatory mechanism has not been elucidated, and no evidence of a physical interaction between YPEL3 and ß -Catenin has been provided so far. Interestingly, the overgrowth of perineurial cells we observe in mz *ypel3* mutants is reminiscent of a dysregulated or defective epithelial-mesenchymal transition. Although WNTs are implicated in distinct aspects of neural development, their role in perineurial glia morphogenesis is unclear. To understand Ypel3 function in motor nerve development, dissection of the role of Ypel3 in the transition of perineurial cells from the neural epithelium to their mesenchymal migratory state and the contribution of Schwann cells to this developmental step will be necessary.

Our interest in *YPEL3* arose from a patient with a variant of unknown significance in this gene who displays unique clinical features including central hypomyelination and peripheral neuropathy. Our aim was to test whether mutations in *YPEL3* could be responsible for these symptoms. Our data show that zebrafish *ypel3* mutants recapitulate the CNS hypomyelination and peripheral nerve defects presented by the patient. Identification of additional patients with *YPEL3* mutations and overlapping phenotypes will provide further confidence that *YPEL3* is a neuropathy gene.

## Materials and methods

### Patient enrollment/consent

The patient was evaluated at the National Institutes of Health Undiagnosed Diseases Program (NIH UDP) [54–56] and was enrolled in the protocol 76-HG-0238, approved by the National Human Genome Research Institute Institutional Review Board. Her parents provided written informed consent.

### Exome sequence/genomic analysis

Whole exome sequencing analysis was performed on the patient, her parents, and two unaffected siblings at the National Institutes of Health Intramural Sequencing Center (NISC). Peripheral whole blood samples were collected for DNA extraction using AutoGen FLEX STAR. Phenol-chloroform purified DNA were prepared enriched for targeted whole exome regions using the TruSeq DNA Sample Prep Kit v1 (Illumina, San Diego, CA) and sequenced on the HiSeq 2000 Sequencing System (Illumina, San Diego, CA) for 101-bp paired-end reads. The sequencing reads then were filtered for quality and aligned to human reference genome NCBI build 37 (hg19) using pipeline developed by the Undiagnosed Diseases Program (UDP), one based on NovoAlign (Novocraft Technologies, Petaling Jaya, Malaysia), and separately, a diploid aligner[57] that was run on a commercial platform (Appistry Inc., St. Louis, MO). Variants were called with HaplotypeCaller and GenotypeGVCFs [58–60]. Variants were annotated using snpEff [61] and a combination of publicly available data sources (ExAC, ESP, 1000Genomes) and internal cohort statistics, manually inspected using the Integrative Genomics Viewer (IGV), and checked for publicly available clinical or functional data in OMIM, HGMD, and PubMed. Variants were interpreted and prioritized based on the clinical relevance of the gene and the pathogenicity of the variants using the ACMG-AMP guidelines [62] or inferred significance based on Mendelian consistency, population frequency, and predicted deleteriousness. Clinical genome sequencing was performed by HudsonAlpha (Huntsville, AL).

### Fish strains

Homozygous WT (*ypel3*^*+/+*^*)*, heterozygous mutants (*ypel3*^*+/b1309*^ and *ypel3*^*+/b1310*^), homozygous mutants (*ypel3* ^*b1309/b1309*^ and *ypel3*^*b1310/b1310*^), maternal-zygotic homozygous mutants (mz *ypel3* ^*b1309/b1309*^ and mz *ypel3*^*b1310/b1310*^), *Tg[olig2*:*kaede]*^*vu85*^ [39]; *Tg Bac[nkx2*.*2*:*mGFP]*^*vu16*^ [17], *Tg[mnx1*:*GFP]*^*ml12*^ [40] and *Tg[sox10(7*.*2)*:*mRFP]*^*vu234*^ [8], and WT strain ABCxTu adult zebrafish were maintained as previously described [63]. Embryos and larvae were staged according to the standard staging series [64], by hours postfertilization (hpf), or days postfertilization (dpf). Adults stocks of *ypel3*^*+/+*^, *ypel3*^*+/b1309*^ and *ypel3*^*+/b1310*^, *ypel3*^*b1309/b1309*^ and *ypel3*^*b1310/b1310*^ were generated by incrossing F2, F3 and F4 heterozygous *ypel3* mutant adults. WT controls (*ypel3*^*+/+*^) used for comparative analysis with mz *ypel3* mutants were obtained by crossing adults *ypel3*^*+/+*^ females with heterozygous adults *ypel3*^*+/b1309*^ or *ypel3*^*+/b1310*^ males. mz *ypel3*^*b1309/b1309*^ and mz *ypel3*^*b1310/b1310*^ were obtained by crossing homozygous *ypel3* ^*b1309/b1309*^ or homozygous *ypel3*^*b1310/b1310*^ females with *ypel3*^*+/b1309*^ and *ypel3*^*+/b1310*^ heterozygous males, respectively. All experimental samples were genotyped. All experimental procedures were approved by the University of Oregon IACUC.

### Protein identity and similarity analysis

The DRSC Integrative Ortholog Prediction Tool (DIOPT) freeware was used to determine protein identity and similarity between human YPEL3 (NP_113665.3), the product encoded by the long human *YPEL3* transcript, and zebrafish Ypel3 (NP_997955.1) proteins. The *YPEL3* gene encodes two transcript variants, a long variant (NM_031477) giving rise to a protein composed of 157 amino acids and a short variant (NM_001145524) giving rise to a protein of 119 amino acid. Only one zebrafish *ypel3* transcript variant has been characterized to date that encodes a protein of 119 amino acids.

### RT-PCR and cloning of *ypel3* coding sequence

RNA was extracted from 26 hpf ABCxTu embryos. PCR was performed after retro-transcription using the SuperScript III First-Strand Synthesis System. Primers used were: *ypel3* forward: 5’-CAAACATCCAGACATGGTGAAG-3’, *ypel3* reverse: 5’-CGGTGACAGCAAGTTAAATACAAA-3’.

### *In situ* hybridization

Embryos and larvae were hybridized with a digoxigenin labeled RNA probe spanning the entire coding sequence of *ypel3*. For wholemount *in situ* hybridization (ISH), we used the previously published protocol [65]. ISH experiments on zebrafish 16 μm thick cryosections were performed using previously described protocols [66], with the following modifications: hybridization and washes were performed at 70°C, and anti-digoxigenin antibody dilution was 1:5000. Images of samples labeled by ISH were acquired using a Zeiss Axioplan2 compound microscope.

### Generation of indel mutant lines using CRISPR

To create the *ypel3* mutant line using the CRISPR/Cas9 system, an RNA guide targeting the 5’- GgtgtttttagGGTGAACGT-3’ sequence at the level of intron3-exon4 junction was designed. Indels were identified using the following primers: Forward 5’-GTAAATGGTTGGGACGTAGCG-3’; Reverse 5’-TTTGGCATCGGTTCTGTTAAA-3’. Two different indels were recovered, *ypel3*^*b1309*^ (p. p.N56Gfs*1) and *ypel3*^*b1310*^ (p.G60Rfs*4). Both F0 carriers were crossed with ABCxTu to generate an F1 stable line. Identified F1 *ypel3* heterozygous mutant fish were outcrossed with a different ABCxTu family to generate an F2 generation. Our phenotypic characterization was performed from the F2 stocks and derived fish families.

### Behavioral assay

Locomotor behavior was assessed in a novel tank test, where individual animals were placed into a custom glass tank measuring 25 cm (width) x 24 cm (height) x 2.5 cm (depth) illuminated with a white LED panel (Environmental Lights). Images were obtained at 10 Hz using a Logitech webcam, and tracking was performed on the fly using a modified form of DaniOPEN [48]. The percentage of time in motion was calculated by dividing the number of frames where the animal’s position moved one third of its body length by the total number of frames acquired. Pauses were defined as a period of time where animals were immobile for a minimum of 1 second.

### Immunolabeling

Labeling of wholemount larvae was performed following our published protocol [67] with minor modifications. Embryos at 52 hpf and 5 dpf and 7 dpf larvae were anesthetized with MS-222 (tricaine-S; Syndel USA) at 4 g/l, fixed in BT fix overnight. Embryos at 52 hpf were permeabilized in 1.5% tween-20 in PBS for 18 hours. Larvae at 5 and 7 dpf were permeabilized in 2.5% tween-20 in PBS for 18 hours or 48 hours, respectively. To perform immunolabeling of sections, samples were genotyped and sorted accordingly to genotypes. WT and mz *ypel3* mutant embryos were embedded in 1% agarose/0.5% agar/5% sucrose and 16 μm cryosections were cut. For single and double immunolabeling, following a 2-hour blocking step (PBS + 0.01% Tween-20 + 5% bovine serum albumin + 5% goat serum), the larvae were incubated overnight at 4°C with primary antibodies. Primary antibodies were rabbit anti-GFP directly coupled with Alexa488 (cat#A-21311, Life technologies, dilution 1:400), rabbit dsRed (cat#632496, Clontech, dilution 1:200), rabbit anti-Kaede (cat#PM012, MBL International, dilution 1:250), rabbit anti-Mbp-b (kind gift from Bruce Appel, dilution 1/100) (Hines et al., 2015). TOPRO (cat#T3605, Life technologies) was reconstituted in PBS following manufacturer’s recommendations. Sections were incubated overnight at 4°C in a 1/1000 diluted TOPRO reconstituted solution. For double labeling of GFP and mRFP, sequential detection was used, during which mRFP signal was amplified first using anti-dsRED followed by the addition of a goat anti-rabbit Alexa 555 secondary antibody (cat# A-21428, Life technologies, dilution 1:500). After 6 washes of 30 minutes each and a 2 hour blocking step, samples were incubated with an anti-rabbit GFP directly coupled to Alexa488. Sections and wholemount samples were imaged in vectashield medium (cat#H-1000, Vectorlabs) using a Zeiss LSM 5 confocal microscope.

### TUNEL labeling

TUNEL staining was performed as previously described with minor modifications [68]. Apoptag plus Peroxidase *In situ* Apoptosis Detection Kit was used (manufacturer). Larvae at 32, 52, and 76 hpf were anesthetized with MS-222, fixed in BT fix overnight, and stored overnight in 100% methanol. After rehydration, the larvae were permeabilized for 20, 30, and 40 minutes in Proteinase K (1 μg/ml), respectively, post-fixed for 20 minutes in BT fix, and incubated in acetone/ethanol mix (1:2) for 15 minutes at -20°C. The samples were equilibrated for 1 hour at room temperature in equilibration buffer, incubated for 90 minutes at 37°C in reaction solution (16 μl TdT enzyme + 38 μl reaction buffer). The reaction was stopped by addition of 200 μl stop buffer overnight at 37°C. The samples were blocked for 2 hours and incubated overnight at 4°C with an anti-DIG coupled to HRP. After several washes in PBST, the reaction product was detected with DAB as a substrate.

### EDU labeling

EdU was added at 50 hpf directly to the Embryo Medium at a final concentration of 0.1 mg/ml. Experimental samples were incubated during 1 hour at 20°C and then rinsed 3 times in Embryo Medium. After incubation, samples were split evenly into two experimental groups, pulse and chase, respectively, and raised at 28.5ºC. Pulse group samples were then fixed at 52 hpf. Chase group samples were fixed at 72 hpf. The Click-iT EdU Imaging Kit (Thermo Fisher Scientific, Waltham, MA) was used to process the EdU label in whole fixed zebrafish prior to antibody staining, according to the manufacturer’s protocols.

### Spinning disk imaging

A Leica SD6000 spinning disk confocal with Borealis illumination technology was used to image live embryos and larvae. Fish from a single clutch were used for imaging only at one stage to prevent stress-induced developmental delay. Larvae were anesthetized in 80 mg/l clove oil (Hilltech), mounted in 0.7% agarose in glass-bottomed Petri dishes, covered with E2 containing clove oil, and, for time-lapse microscopy, imaged at 25-minute intervals. Larvae imaged in this manner appeared similar to staged, non-imaged controls. Videos were constructed from z-projections using Metamorph imaging software (Molecular Devices).

### Metabolomics

Prior to extraction, 450 individual zebrafish larvae were pooled to provide enough material for metabolomics analysis (n = 5 for WT and mutant). Larvae were transferred to 2.0 mL Sorenson low-binding microcentrifuge tubes. Cellular homogenization was conducted by adding 0.1 μl of 0.1 mm Zirconica/silica beads to each tube then processing with a Bullet Blender (BB50-DX) at speed 10 for 3 minutes as suggested by Next Advance protocols. The homogenization step was repeated. After the bead beating, the tubes were visually inspected for complete homogenization. Metabolites and lipids were extracted using a modified Folch extraction (MPLEx) [69,70]. The extracted polar metabolite layer was, dried *in vacuo*, and stored at -20˚C until Mass Spectroscopy (MS) sample preparation. The total lipid extract was reconstituted in 500 μl of 2:1 chloroform/methanol and stored at -20˚C until LC-MS analysis.

Prior to LC-MS analysis, lipid extracts were dried and then reconstituted in 10 μl chloroform and 490 μl of methanol. LC-MS/MS parameters and lipid identifications are outlined in [71]. To facilitate quantification of lipids, a list of lipids identified from the MS/MS data was created, including lipid name, observed *m/z*, and retention time. Lipid features from each analysis were then aligned based on their *m/z* and retention time using MZmine 2 [72]. Aligned features were manually verified and peak apex intensity values were exported for subsequent statistical analysis.

Dried metabolite extracts were chemically derivatized using a modified protocol used to create the FiehnLib [73] as described [74]. Metabolite identifications were manually validated with peak intensity values used for statistical analysis.

Metabolomics and lipidomics were analyzed separately and data were log2 transformed prior to further processing. Negative and positive mode lipidomics data were processed separately. To identify any potential outlying samples, a robust Mahalanobis distance (rMd) filter was applied [75] and analyzed on the basis of correlation, median absolute deviation, and skew. One biological outlier was identified (WT replicate 4) in the metabolomics data and confirmed via Pearson correlation between the samples, and was therefore removed from further processing. All data were normalized using global median centering. Metabolites and lipids were evaluated with Analysis of Variance (ANOVA) with a Holm p-value test correction [76].

## Supporting information

S1 Fig YPEL3 is evolutionarily conserved and expressed within the developing CNS and in the motor nerve. (**A**) Alignment of human and zebrafish YPEL3 protein variants. The *YPEL3* gene encodes two transcript variants: a long variant (NM_031477) that gives rise to a protein composed of 157 amino acids (NP_113665.3) and a short variant (NM_001145524) that encodes a protein of 119 amino acids (NP_001138996.10. One zebrafish *ypel3* transcript variant (NM_212790.1) has been characterized to date. It encodes a protein of 119 amino acids (NP_997955.1). TV1: Long human YPEL3 protein variant. TV2: Short human YPEL3 protein variant. danio: Ypel3 zebrafish protein. Blue arrowhead indicates the location of Val92 in the long human isoform. (**B**) Section of 80 hpf zebrafish larva showing ypel3 expression at the level of the spinal cord and motor nerve (red arrows). (**C**) Section of the same specimen at a more rostral level of the spinal cord. Note the absence of ypel3 in the ventral medial region. Scale bar: 25 μm.
(TIF)

S2 Fig *ypel3*^*b1309*^ and *ypel3*^*b1310*^ CRISPR engineered alleles are equivalent to the *YPEL3* variant. (**A**) Zebrafish mutant alleles created by CRISPR/Cas9 technology. Two alleles were generated using the same single guide RNA. Both *ypel3*^*b1309*^ and *ypel3*^*b1310*^ are frameshift mutations, p.Asp56Glyfs*1 and p.Gly60Argfs*4, respectively. (**B**) Schematic representations of WT human and zebrafish YPEL3 variants. WT YPEL3: WT human short isoform of 119 amino acids (aa). WT Ypel3: wild-type zebrafish isoform of 119 aa. (**C**) Schematic representation of mutant YPEL3 variants based on the short isoform annotations. UDN variant: predicted YPEL3 variant found in the affected person. B1309, B1310: mutant zebrafish protein variants. Light blue: endogenous amino acids. Green: Exogenous amino acids introduced by the frameshift mutation.
(TIF)

S3 Fig Loss of Ypel3 does not affect motor axon development. (**A-B**) Quantification of motor nerve width or thickness in WT and mz *ypel3* mutants at 26 (**A**) and 52 (**B**) hpf. (**C-D**) Quantification of average motor nerve length (**C**) and distance between motor nerves in WT and mz *ypel3* mutants at 80 hpf.
(TIF)

S4 Fig Loss of Ypel3 transiently depletes OPC population and results in oligodendrocyte filopodial defects. (**A**) Average number of GFP+, mRFP+ double positive oligodendrocytes per somite as a function of developmental stage. (**B**) Average number of filopodia per myelinating oligodendrocyte. (**C**) Average filopodial length.
(TIF)

S5 Fig *sox10+ olig2+* oligodendrocyte population recovers in mz *ypel3* mutants. (**A, B**) Lateral views at the level of the spinal cord from somites 8 to 11 of 80 hpf larvae. WT and mz *ypel3* mutant embryos were photoconverted at 26 hpf and then imaged at 80 hpf. As expected, primary motoneurons are labelled magenta (arrowhead). Newly added cells are labeled green (Tg[*olig2*:*kaede*]). Oligodendrocytes are colabeled with Tg[*olig2*:*kaede*]) and Tg[*sox10*:*mRFP*] (arrows in A and B). (**C**) Quantification of the average total number of *sox10+*, *olig2+* oligodendrocytes at 80 hpf in WT and mz *ypel3*^*-/-*^ mutants. (**D**) Quantification of the average total number of *nkx2.2+*, *sox10+* oligodendrocytes at 7 dpf.
(TIF)

S6 Fig Quantification of the number of sox10:mRFP cells at the level of the motor nerves at 28 hpf. (TIF)

S1 Table Bioinformatically relevant variants identified through exome sequencing. a, nomenclature based on GRCh37 (hg19). CADD, Combined Annotation Dependent Depletion (https://cadd.gs.washington.edu/). PolyPhen2 (http://genetics.bwh.harvard.edu/pph2/). SIFT, Sorting Intolerant From Tolerant (https://sift.bii.a-star.edu.sg/). gnomAD, Genome Aggregation Database (https://gnomad.broadinstitute.org/).
(DOCX)

S2 Table Members of the Undiagnosed Diseases Network. (DOCX)

S1 Video Ypel3 is required for OPC and perineurial glia development. Wild type. Time-lapse video from 50 to 62 hpf. Each frame is a projection of a 50 μm deep Z-stack of lateral views acquired every 25 minutes using a spinning disk microscope. Dark yellow arrows indicate OPCs. Green arrows indicate perineurial glial cells that have exited the CNS. Scale bar: 20 μm.
(AVI)

S2 Video Ypel3 is required for OPC and perineurial glia development. mz *ypel3* mutant. Time-lapse video from 50 to 62 hpf. Each frame is a projection of a 50 μm deep Z-stack of lateral views acquired every 25 minutes using a spinning disk microscope. Dark yellow arrows indicate OPCs. Green arrows indicate perineurial glial cells that have exited the CNS. Scale bar: 20 μm.
(AVI)
