# Correction: *yippee like 3 (ypel3)* is a novel gene required for myelinating and perineurial glia development

**DOI:** 10.1371/journal.pgen.1009156

**Published:** 2020-10-26

**Authors:** Bernardo Blanco-Sánchez, Aurélie Clément, Sarah J. Stednitz, Jennifer Kyle, Judy L. Peirce, Marcie McFadden, Jeremy Wegner, Jennifer B. Phillips, Ellen Macnamara, Yan Huang, David R. Adams, Camilo Toro, William A. Gahl, May Christine V. Malicdan, Cynthia J. Tifft, Erika M. Zink, Kent J. Bloodsworth, Kelly G. Stratton, Undiagnosed Diseases Network, David M. Koeller, Thomas O. Metz, Philip Washbourne, Monte Westerfield

The third author’s name is spelled incorrectly. The correct name is: Sarah J. Stednitz
