# Supplementary figures and images for: yippee like 3 (ypel3) is a novel gene required for myelinating and perineurial glia development
Source: PLoS Genet. 2020 Jun 16;16(6):e1008841. doi: 10.1371/journal.pgen.1008841 (PMC7319359; doi:10.1371/journal.pgen.1008841)

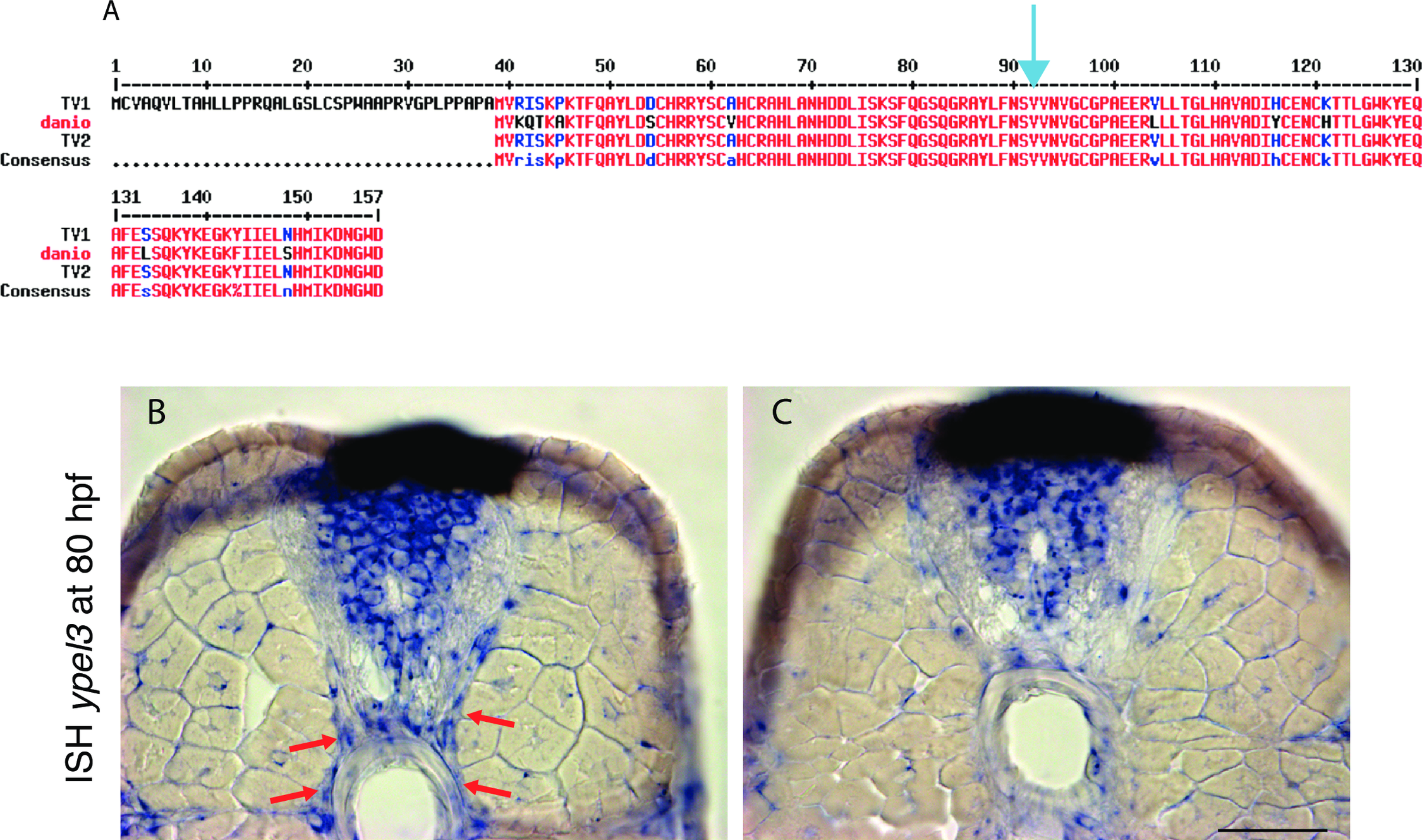

Supplement: S1 Fig — (A) Alignment of human and zebrafish YPEL3 protein variants. The YPEL3 gene encodes two transcript variants: a long variant (NM_031477) that gives rise to a protein composed of 157 amino acids (NP_113665.3) and a short variant (NM_001145524) that encodes a protein of 119 amino acids (NP_001138996.10. One zebrafish ypel3 transcript variant (NM_212790.1) has been characterized to date. It encodes a protein of 119 amino acids (NP_997955.1). TV1: Long human YPEL3 protein variant. TV2: Short human YPEL3 protein variant. danio: Ypel3 zebrafish protein. Blue arrowhead indicates the location of Val92 in the long human isoform. (B) Section of 80 hpf zebrafish larva showing ypel3 expression at the level of the spinal cord and motor nerve (red arrows). (C) Section of the same specimen at a more rostral level of the spinal cord. Note the absence of ypel3 in the ventral medial region. Scale bar: 25 μm. (TIF) [file pgen.1008841.s001.tif]

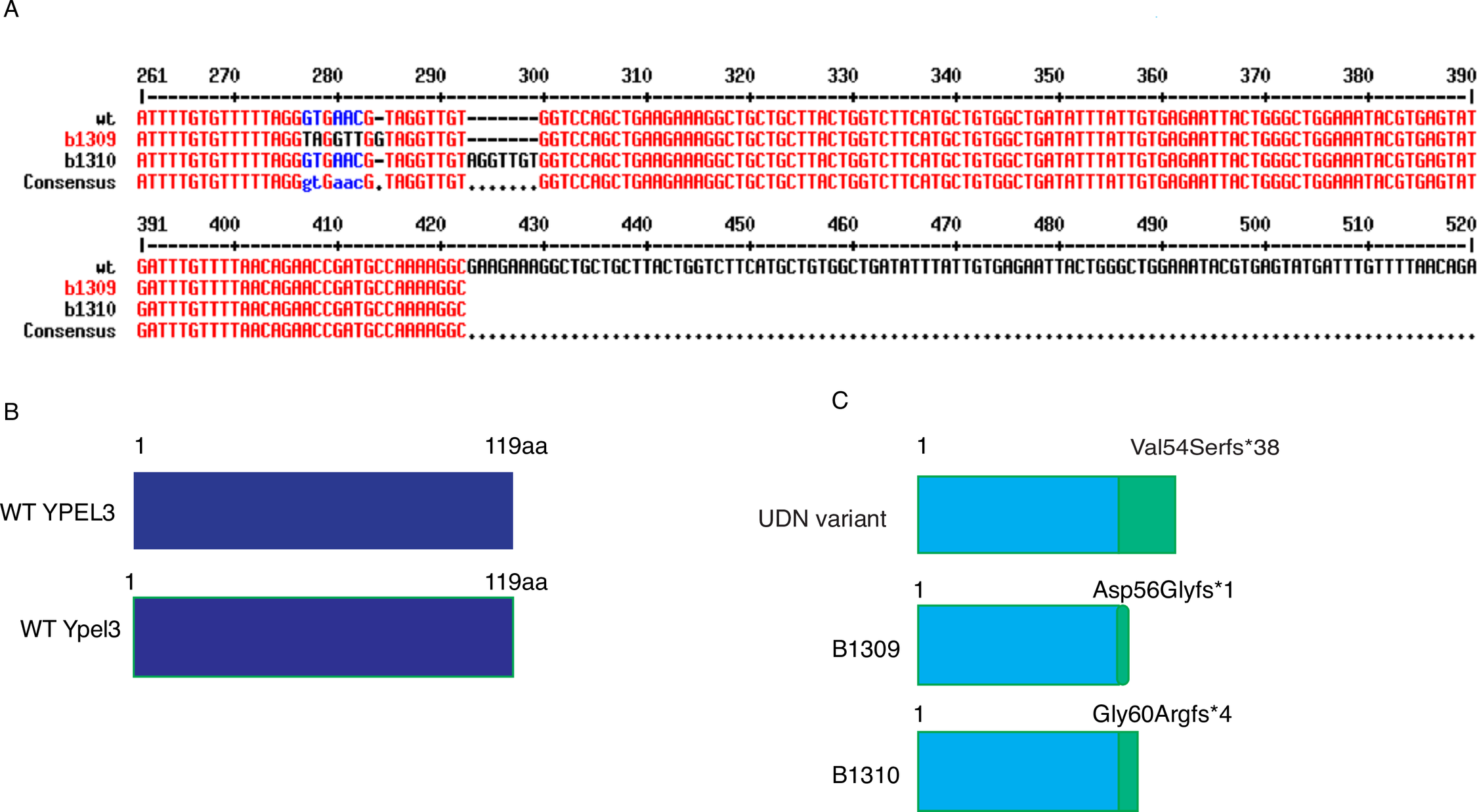

Supplement: S2 Fig — (A) Zebrafish mutant alleles created by CRISPR/Cas9 technology. Two alleles were generated using the same single guide RNA. Both ypel3b1309 and ypel3b1310 are frameshift mutations, p.Asp56Glyfs*1 and p.Gly60Argfs*4, respectively. (B) Schematic representations of WT human and zebrafish YPEL3 variants. WT YPEL3: WT human short isoform of 119 amino acids (aa). WT Ypel3: wild-type zebrafish isoform of 119 aa. (C) Schematic representation of mutant YPEL3 variants based on the short isoform annotations. UDN variant: predicted YPEL3 variant found in the affected person. B1309, B1310: mutant zebrafish protein variants. Light blue: endogenous amino acids. Green: Exogenous amino acids introduced by the frameshift mutation. (TIF) [file pgen.1008841.s002.tif]

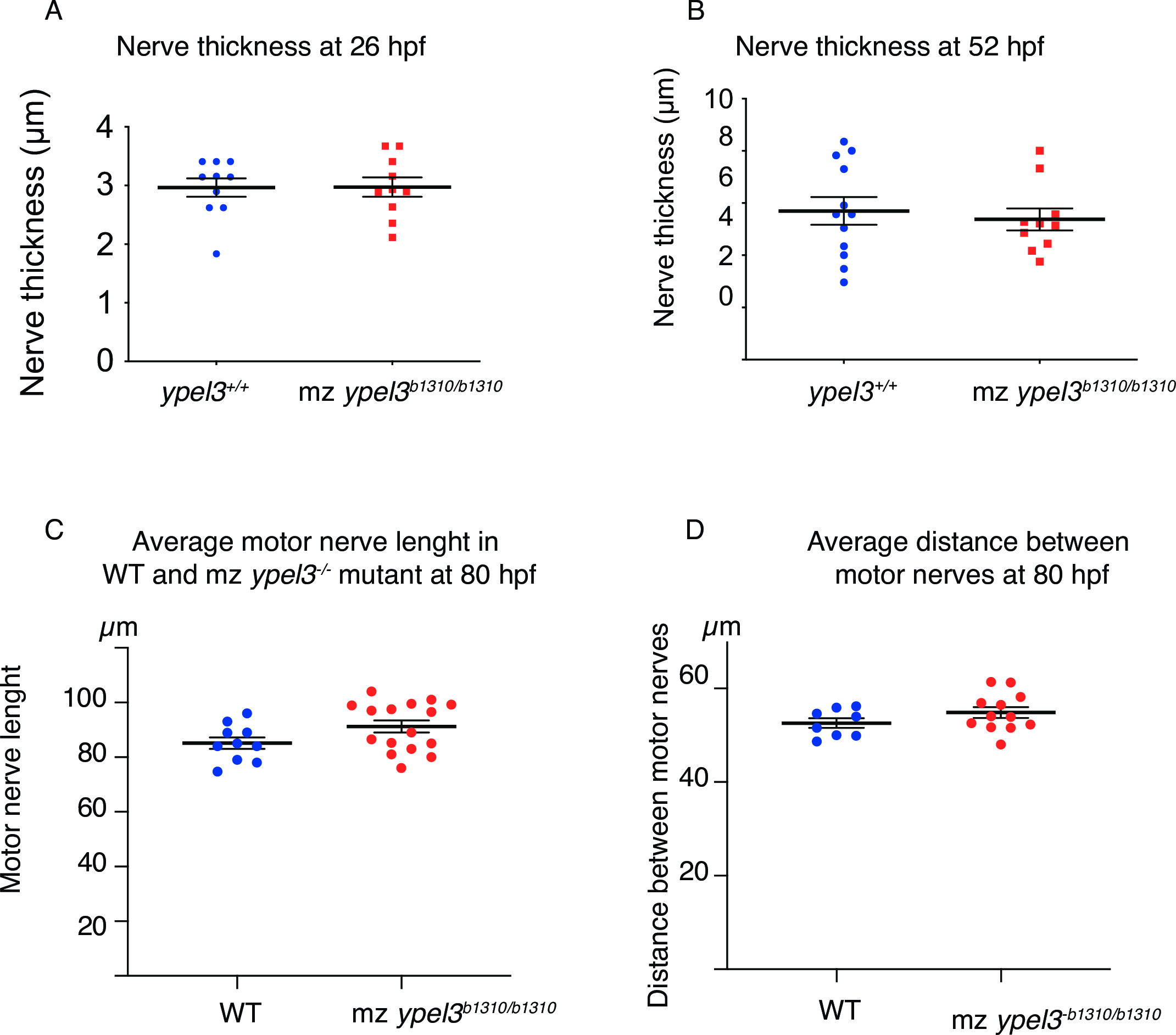

Supplement: S3 Fig — (A-B) Quantification of motor nerve width or thickness in WT and mz ypel3 mutants at 26 (A) and 52 (B) hpf. (C-D) Quantification of average motor nerve length (C) and distance between motor nerves in WT and mz ypel3 mutants at 80 hpf. (TIF) [file pgen.1008841.s003.tif]

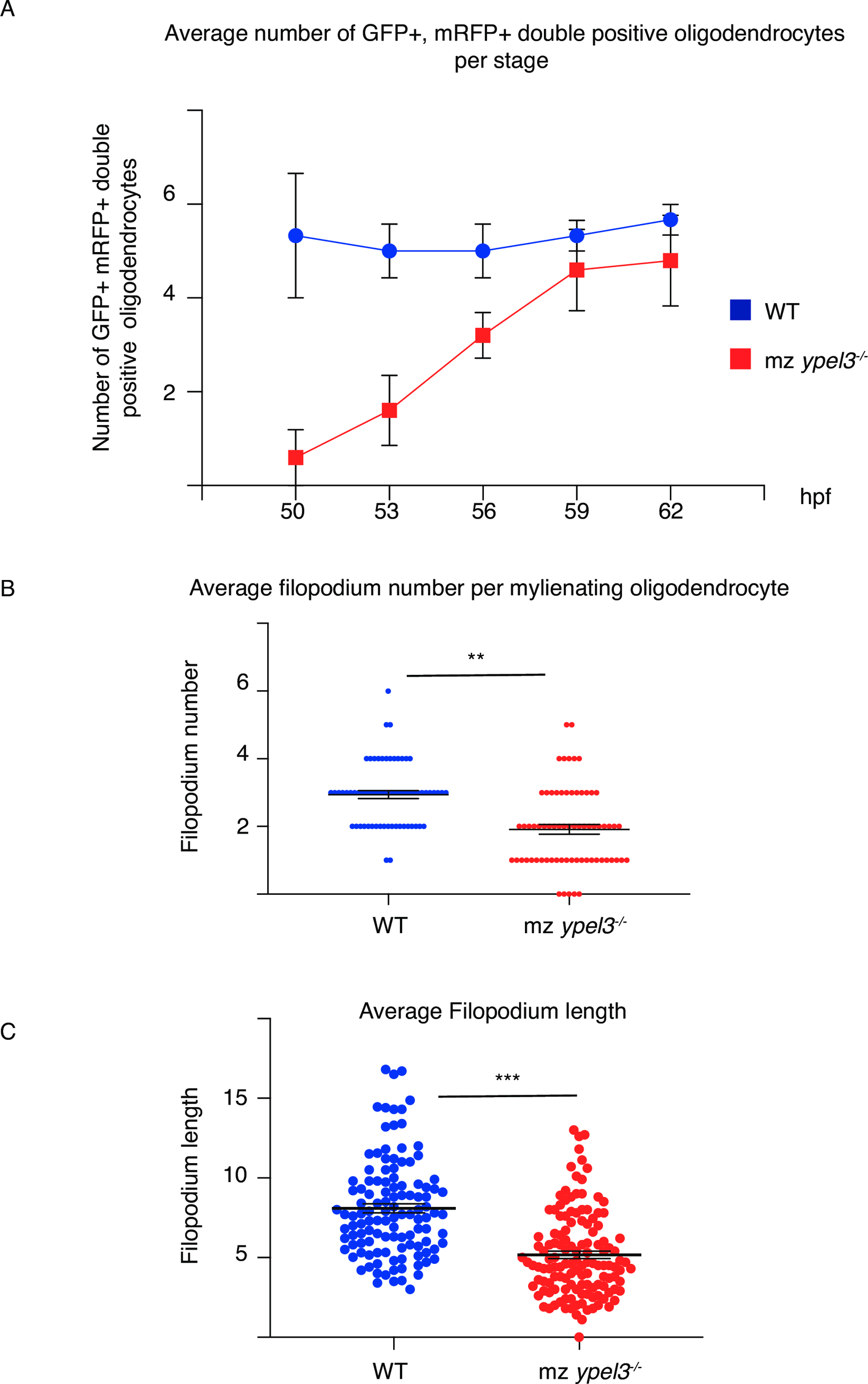

Supplement: S4 Fig — (A) Average number of GFP+, mRFP+ double positive oligodendrocytes per somite as a function of developmental stage. (B) Average number of filopodia per myelinating oligodendrocyte. (C) Average filopodial length. (TIF) [file pgen.1008841.s004.tif]

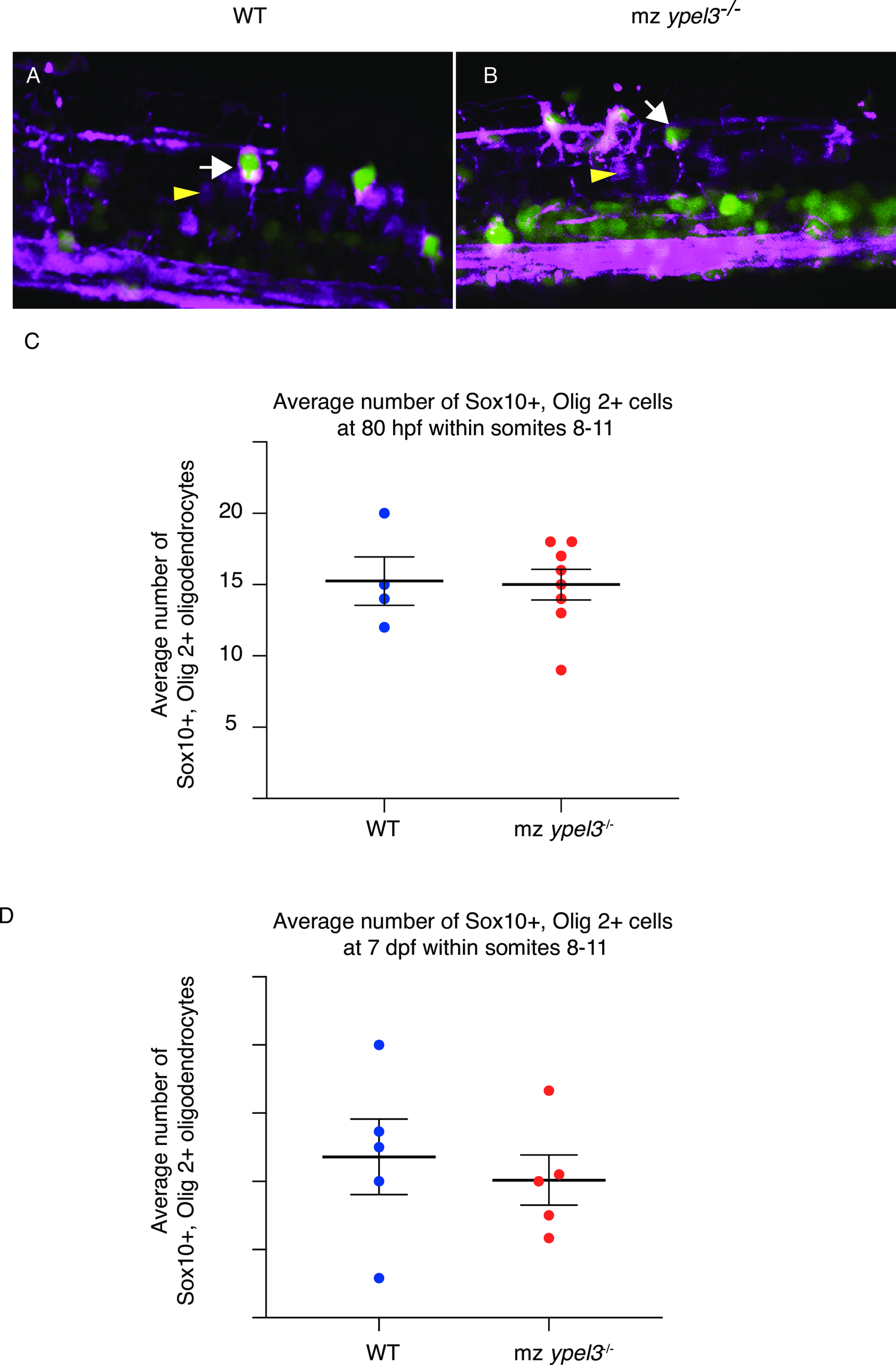

Supplement: S5 Fig — (A, B) Lateral views at the level of the spinal cord from somites 8 to 11 of 80 hpf larvae. WT and mz ypel3 mutant embryos were photoconverted at 26 hpf and then imaged at 80 hpf. As expected, primary motoneurons are labelled magenta (arrowhead). Newly added cells are labeled green (Tg[olig2:kaede]). Oligodendrocytes are colabeled with Tg[olig2:kaede]) and Tg[sox10:mRFP] (arrows in A and B). (C) Quantification of the average total number of sox10+, olig2+ oligodendrocytes at 80 hpf in WT and mz ypel3-/- mutants. (D) Quantification of the average total number of nkx2.2+, sox10+ oligodendrocytes at 7 dpf. (TIF) [file pgen.1008841.s005.tif]

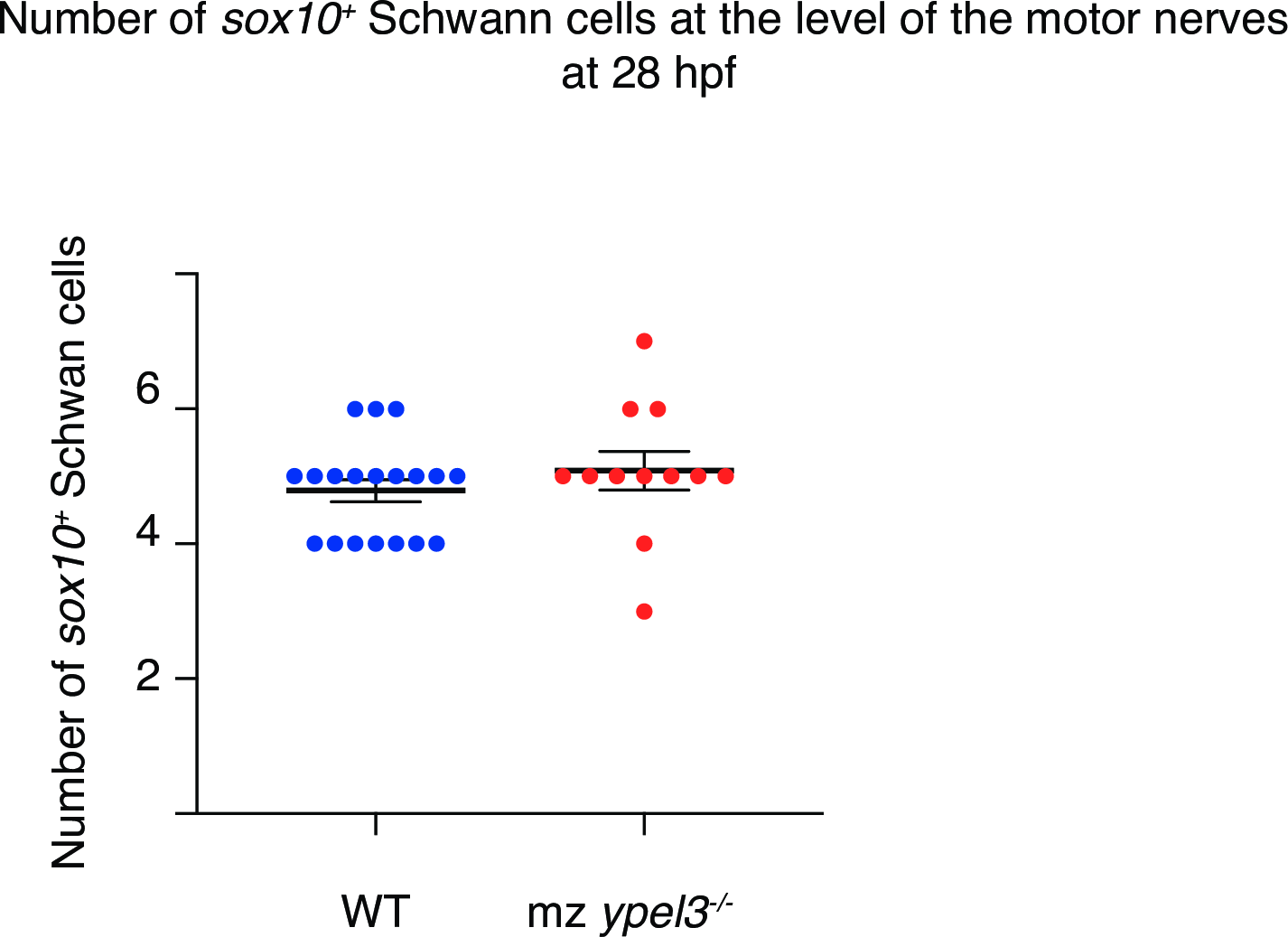

Supplement: S6 Fig — (TIF) [file pgen.1008841.s006.tif]
