# Supplementary material for: yippee like 3 (ypel3) is a novel gene required for myelinating and perineurial glia development
Source: PLoS Genet. 2020 Jun 16;16(6):e1008841. doi: 10.1371/journal.pgen.1008841 (PMC7319359; doi:10.1371/journal.pgen.1008841)
Supplement: S1 Table — a, nomenclature based on GRCh37 (hg19). CADD, Combined Annotation Dependent Depletion (https://cadd.gs.washington.edu/). PolyPhen2 (http://genetics.bwh.harvard.edu/pph2/). SIFT, Sorting Intolerant From Tolerant (https://sift.bii.a-star.edu.sg/). gnomAD, Genome Aggregation Database (https://gnomad.broadinstitute.org/). (DOCX) [file pgen.1008841.s007.docx]

**Supplementary table 1: Gene variants identified by exome sequencing**

| **Gene** | **Inheritance / Allele** | **Parent of origin** | **Nucleotide Change^a^** | **Amino Acid Change** | **CADD (v1.3, C), PolyPhen (P), SIFT (S)** | **gnomAd frequency** | **gnomAd frequency (European)** | **Disease Association** | **Gene Info** | **Comments** |
| --- | --- | --- | --- | --- | --- | --- | --- | --- | --- | --- |
| *TNFRSF8* | *De novo* | None | chr1:12198373del; NM_001243.4: c.1423del | p.Val475Trpfs*46 | C:24.3 | 0 | 0 | None | Tumor Necrosis Factor Receptor Superfamily, Member 8: a member of the TNF-receptor superfamily, it is expressed by activated, but not by resting, T and B cells. TRAF2 and TRAF5 can interact with this receptor, and mediate the signal transduction that leads to the activation of NF-kappaB. This receptor is a positive regulator of apoptosis, and also has been shown to limit the proliferative potential of autoreactive CD8 effector T cells and protect the body against autoimmunity. | Deletion of gene in mice leads to enlarged thymus and increased thymocyte number. A frameshift deletion in the same exon observed in 1 instance (South Asian population). |
| *TG* | Compound heterozygous | Paternal | chr8:133894816G>A; NM_003235.4:c.848G>A | p.Arg283Glu | C:34 P:probably damaging S:deleterious | 0.0006687 | 0.001455 | Thyroid dyshormonogenesis | Thyroglobulin (Tg) is a glycoprotein homodimer produced predominantly by the thryroid gland. It acts as a substrate for the synthesis of thyroxine and triiodothyronine as well as the storage of the inactive forms of thyroid hormone and iodine. Thyroglobulin is secreted from the endoplasmic reticulum to its site of iodination, and subsequent thyroxine biosynthesis, in the follicular lumen. | Patient had no thyroid abnormalities |
|  |  | Maternal | chr8:133895127C>T; NM_003235.4:c.958C>T | p.Arg320Cys | C:23.2 P:possibly damaging S:deleterious | 0.0005096 | 0.000449 |  |  |  |
| *FOXRED2* | Compound heterozygous | Maternal | chr22:36900186G>C; NM_001102371.1: c.1008C>G | p.Ile336Met | C:25.3 P:probably damaging S:deleterious | 0.001369 | 0.001632 | None | FAD-Dependent Oxidoreductase Domain Containing 2: probable flavoprotein which may function in endoplasmic reticulum associated degradation (ERAD). May bind non-native proteins in the endoplasmic reticulum and target them to the ubiquitination machinery for subsequent degradation | Deletion of this gene in mice leads to abnormality in limb grasp |
|  |  | Paternal | chr22:36900220T>C; NM_001102371.1: c.974A>G | NM_024955: Asn325Ser | C:14.71 P:benign S:tolerated | 0.000028 | 0.00004646 |  |  |  |
| *YPEL3* | DeNovo / Mono | None | chr16:30106424A>AT | p.Val92Glyfs*38 | C:35 | 0 | 0 | None | Yippee-Like 3. Nothing much is known about this gene. Protein is upregulated by p53 activation following p53-inducing DNA damage. |  |
|  |  |  |  |  |  |  |  |  |  |  |
| Bioinformatically relevant variants identified through exome sequencing. a, nomenclature based on GRCh37 (hg19). CADD, Combined Annotation Dependent Depletion (https://cadd.gs.washington.edu/). PolyPhen2 (http://genetics.bwh.harvard.edu/pph2/). SIFT, Sorting Intolerant From Tolerant (https://sift.bii.a-star.edu.sg/). gnomAD, Genome Aggregation Database (https://gnomad.broadinstitute.org/). | | | | | | | | | | |
